# Control of astrocytic Ca^2+^ signaling by nitric oxide-dependent S-nitrosylation of Ca^2+^ homeostasis modulator 1 channels

**DOI:** 10.1186/s40659-024-00503-3

**Published:** 2024-04-30

**Authors:** Mariela Puebla, Manuel F. Muñoz, Mauricio A. Lillo, Jorge E. Contreras, Xavier F. Figueroa

**Affiliations:** 1 Departamento de Fisiología, Facultad de Ciencias Biológicas, Pontificia Universidad Católica de Chile, 8330025 Santiago, Chile; 2 Department of Physiology and Membrane Biology, University of California Davis, Davis, CA USA; 3 Department of Pharmacology, Physiology and Neuroscience, New Jersey Medical School, Rutgers University, Newark, NJ USA

**Keywords:** Nitric oxide, Astrocytes, Ca^2+^ signaling, CALHM1 channels, ATP release, Connexin 43 hemichannels, Pannexin-1 channels

## Abstract

**Background:**

Astrocytes Ca^2+^ signaling play a central role in the modulation of neuronal function. Activation of metabotropic glutamate receptors (mGluR) by glutamate released during an increase in synaptic activity triggers coordinated Ca^2+^ signals in astrocytes. Importantly, astrocytes express the Ca^2+^-dependent nitric oxide (NO)-synthetizing enzymes eNOS and nNOS, which might contribute to the Ca^2+^ signals by triggering Ca^2+^ influx or ATP release through the activation of connexin 43 (Cx43) hemichannels, pannexin-1 (Panx-1) channels or Ca^2+^ homeostasis modulator 1 (CALHM1) channels. Hence, we aim to evaluate the participation of NO in the astrocytic Ca^2+^ signaling initiated by stimulation of mGluR in primary cultures of astrocytes from rat brain cortex.

**Results:**

Astrocytes were stimulated with glutamate or t-ACPD and NO-dependent changes in [Ca^2+^]_i_ and ATP release were evaluated. In addition, the activity of Cx43 hemichannels, Panx-1 channels and CALHM1 channels was also analyzed. The expression of Cx43, Panx-1 and CALHM1 in astrocytes was confirmed by immunofluorescence analysis and both glutamate and t-ACPD induced NO-mediated activation of CALHM1 channels via direct S-nitrosylation, which was further confirmed by assessing CALHM1-mediated current using the two-electrode voltage clamp technique in Xenopus oocytes. Pharmacological blockade or siRNA-mediated inhibition of CALHM1 expression revealed that the opening of these channels provides a pathway for ATP release and the subsequent purinergic receptor-dependent activation of Cx43 hemichannels and Panx-1 channels, which further contributes to the astrocytic Ca^2+^ signaling.

**Conclusions:**

Our findings demonstrate that activation of CALHM1 channels through NO-mediated S-nitrosylation in astrocytes in vitro is critical for the generation of glutamate-initiated astrocytic Ca^2+^ signaling.

**Supplementary Information:**

The online version contains supplementary material available at 10.1186/s40659-024-00503-3.

## Background

Brain function relies on complex interactions in the neural network through inter- and intracellular signaling cascades in which astrocytes, a subtype of glial cells, play an essential role. Astrocytes are multifunctional cells that are involved in several aspects of brain function, such as neuronal metabolism, synaptogenesis, homeostasis of the extracellular milieu and regulation of blood–brain barrier permeability [1, 2]. In addition, the functional coordination of local blood flow according to the changes in neuronal activity (i.e. neurovascular coupling) is also mediated by astrocytes [3–6]. Interestingly, changes in intracellular free Ca^2+^ concentration ([Ca^2+^]_i_) have emerged as the most prominent signaling in astrocytic-regulated brain functions [7–14].

Astrocytes express a wide variety of receptors, which can be activated by neurotransmitters released during an increase in neuronal activity [15–17]. As glutamate is the major excitatory signal in the central nervous system (CNS), the response to this neurotransmitter has typically been analyzed in the study of astrocyte Ca^2+^ signaling [18–21]. In this context, glutamate-initiated astrocytic Ca^2+^ signals are mainly mediated by the activation of metabotropic glutamate receptors (mGluR), which leads to an increase in [Ca^2+^]_i_ by the release of Ca^2+^ from the intracellular Ca^2+^ stores through activation of an inositol [1, 4, 5]-triphosphate (IP_3_)-mediated pathway [17, 22–24]. More recently; however, the activation of an ATP release-initiated purinergic signaling has also been proposed to contribute to the astrocytic Ca^2+^ signaling [25, 26].

Astrocytes are functionally associated with multiple neuronal synapses and a change in focal neuronal activity is sensed by several astrocytes [27, 28]. Then, the astrocyte Ca^2+^ signaling must be coordinated between all astrocytes involved in a particular synaptic transmission to integrate the response. It is thought that connexin (Cx)-mediated communication plays an important role in the coordination of astrocyte Ca^2+^ signaling [12, 29–31]. Cxs are protein subunits that form the intercellular channels known as gap junction, which directly connect the cytoplasm of two adjacent cells, allowing intercellular transfer of current and small solutes (< 1.4 nm of diameter), such as ions and second messengers (e.g. Ca^2+^ and IP_3_). The assembly of six Cxs forms a hemichannel, which docks with a hemichannel from the apposed plasma membrane of a neighboring cell to form a gap junction channel [32–34]. Undocked hemichannels at the plasma membrane; however, can also operate independently to provide communication between the intra and extracellular compartments allowing influx of ions or release of paracrine/autocrine signals, such as ATP [35–38]. In addition to Cxs, another protein family of three members, termed pannexins (Panx-1, Panx-2 and Panx-3), also form membrane channels with similar permeability properties to those described for hemichannels [39–41]. In astrocytes, Cx30, Cx43 and Panx-1 form the most prominent large-pore channels described to provide a pathway for ATP release [36, 42–45]. It should be noted; however, that recently discovered channels, such as Ca^2+^ homeostasis modulator 1 (CALHM1), have emerged as important transmembrane pathways for ATP release [46–49]. Although the presence of CALHM1 has been demonstrated in CNS, the expression of these channels has not been confirmed in astrocytes.

Opening of Cx43 hemichannels and Panx-1 channels is controlled by intracellular Ca^2+^ and their activation may further contribute to amplify and coordinate the astrocyte Ca^2+^ signals [50–53]. Importantly, Cx43 hemichannels are also activated by nitric oxide (NO)-mediated S-nitrosylation of this Cx protein [32, 54]. In contrast, the effect of NO on Panx-1-formed channels is controversial [55, 56] and the participation of this signaling mechanism on the control of CALHM1 channels has not been studied. Interestingly, all three isoforms of the enzyme that generates NO have been described to be expressed in astrocytes: endothelial NO synthase (eNOS), neuronal NO synthase (nNOS) and inducible NO synthase (iNOS) [57–63]. As activation of eNOS and nNOS depends on an increase in [Ca^2+^]_i_, an initial Ca^2+^-dependent NO production may be involved in the regulation of the metabotropic receptor-triggered astrocytic Ca^2+^ signaling through providing a pathway for Ca^2+^ influx or ATP release via Cx43 hemichannels or Panx-1 channels.

In this work, we analyzed the involvement of astrocytic NO production in the regulation of the Ca^2+^ signaling triggered by glutamate in primary cultures of astrocytes. Our findings indicate that stimulation of astrocyte mGluRs is coupled to NO-mediated activation of CALHM1 channels via direct S-nitrosylation, which in turn leads to ATP release. The subsequent ATP-dependent purinergic receptor stimulation induces the opening of Cx43 hemichannels and Panx-1 channels, which contributes to the progress of the astrocytic Ca^2+^ signaling.

## Results

**Fig. 1:**
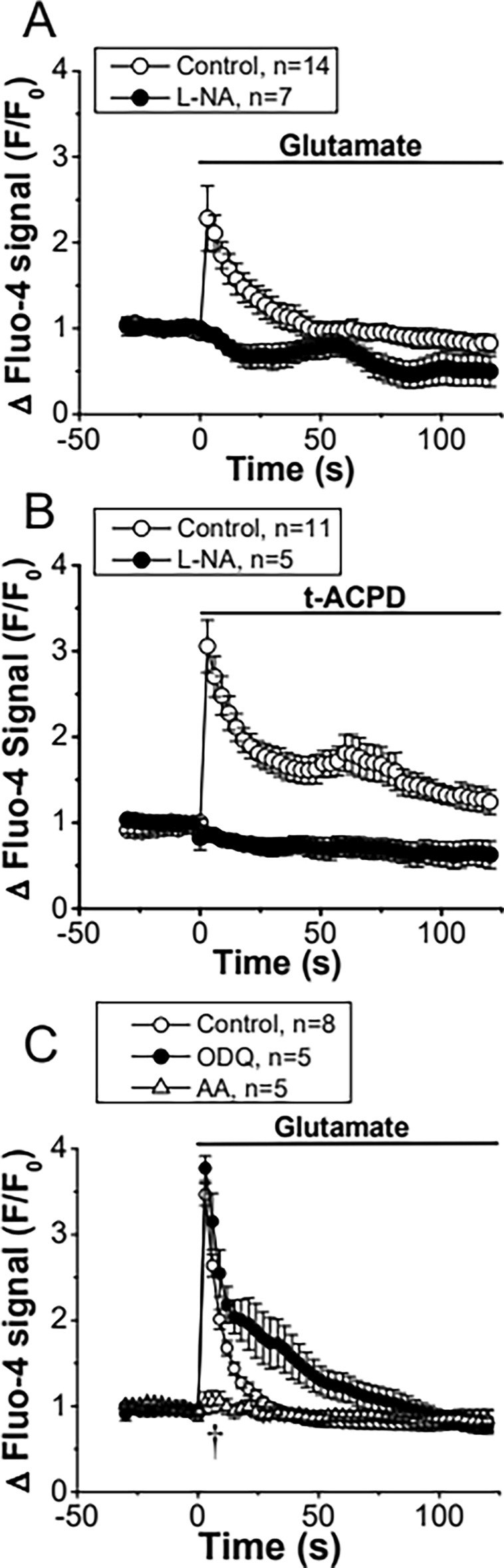
The Ca^2+^ signaling initiated by the activation of metabotropic glutamate receptors (mGluRs) in primary cultures of astrocytes relies on a cGMP-independent NO-mediated pathway. **A** Time course of the increase in [Ca^2+^]_i_ observed in primary cultures of astrocytes in response to 10 µM glutamate before (control) and after blocking NO production with 100 µM N^ω^-nitro-L-arginine (L-NA). **B** Time course of the Ca^2+^ signaling evoked by the stimulation of mGluRs with t-ACPD in control conditions and in the presence of L-NA. Horizontal bars indicate the period of stimulation. **C**, Maximal increment in [Ca^2+^]_i_ induced by glutamate in control conditions and after treating the astrocyte cultures with 10 µM ODQ, a soluble guanylyl cyclase inhibitor, or 50 µM ascorbic acid (AA), a potent reducer. Values are means ± SEM. *P < 0.05 vs Control by two-way ANOVA. ^†^P < 0.05 vs Control by one-way ANOVA plus Bonferroni post hoc test

The mechanisms involved in the regulation of astrocyte-mediated signaling initiated by the activation of glutamate receptors were directly evaluated by measuring the changes in [Ca^2+^]_i_ in primary cultures of astrocytes using Fluo-4. Ca^2+^ levels were stable in basal conditions and application of 10 µM glutamate or 150 µM trans-1-amino-1,3-cyclopentanedicarboxylic acid (t-ACPD), an agonist of mGluRs, activated a rapid increase in the Fluo-4-generated fluorescence signal that reached a peak 3 to 6 s after the stimulation (Fig. 1A and B). While the Ca^2+^ signal-triggered by glutamate was transient and returned to the control level within ~ 40 to 50 s (Fig. 1A), the response to t-ACPD only dropped to a plateau of lower magnitude that slowly declined along the time (Fig. 1B).

### Glutamate-activated Ca^2+^ signaling depends on NO production

**Fig. 2:**
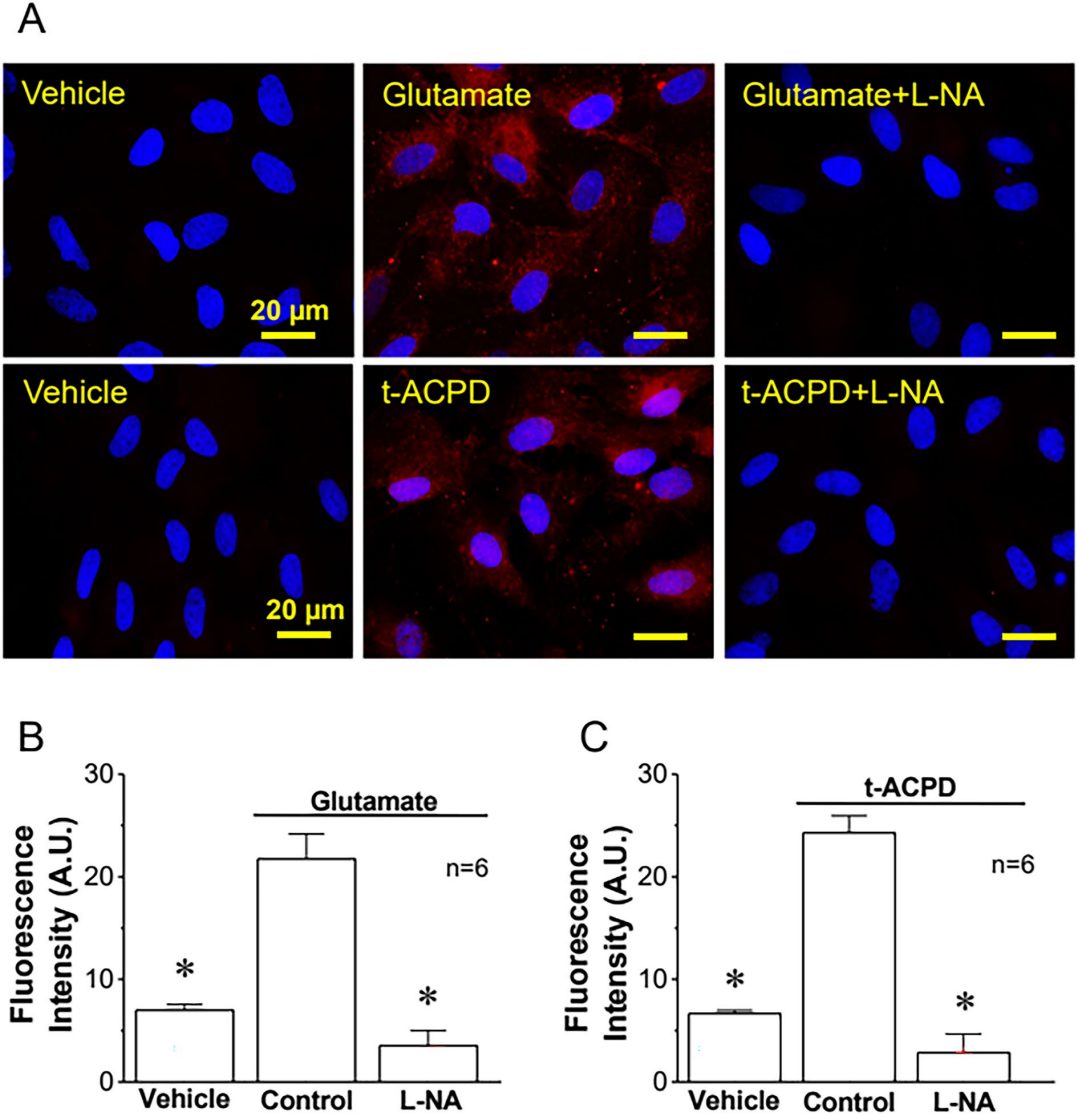
Activation of metabotropic glutamate receptors (mGluRs) in primary cultures of astrocytes evokes NO-dependent S-nitrosylation. **A** Immunofluorescence analysis of total protein S-nitrosylation in primary cultures of astrocytes in response to the stimulation with glutamate or t-ACPD in control conditions and after the inhibition of NO production with 100 µM N^ω^-nitro-L-arginine (L-NA). The effect of the vehicle of glutamate and t-ACPD is also shown. **B** and **C** Fluorescence intensity analysis of the protein S-nitrosylation observed in the experiments shown in **A**. Changes in fluorescence intensity are expressed in arbitrary units (A.U.). Values are means ± SEM. *P < 0.05 vs Control by one-way ANOVA plus Bonferroni post hoc test

**Fig. 3:**
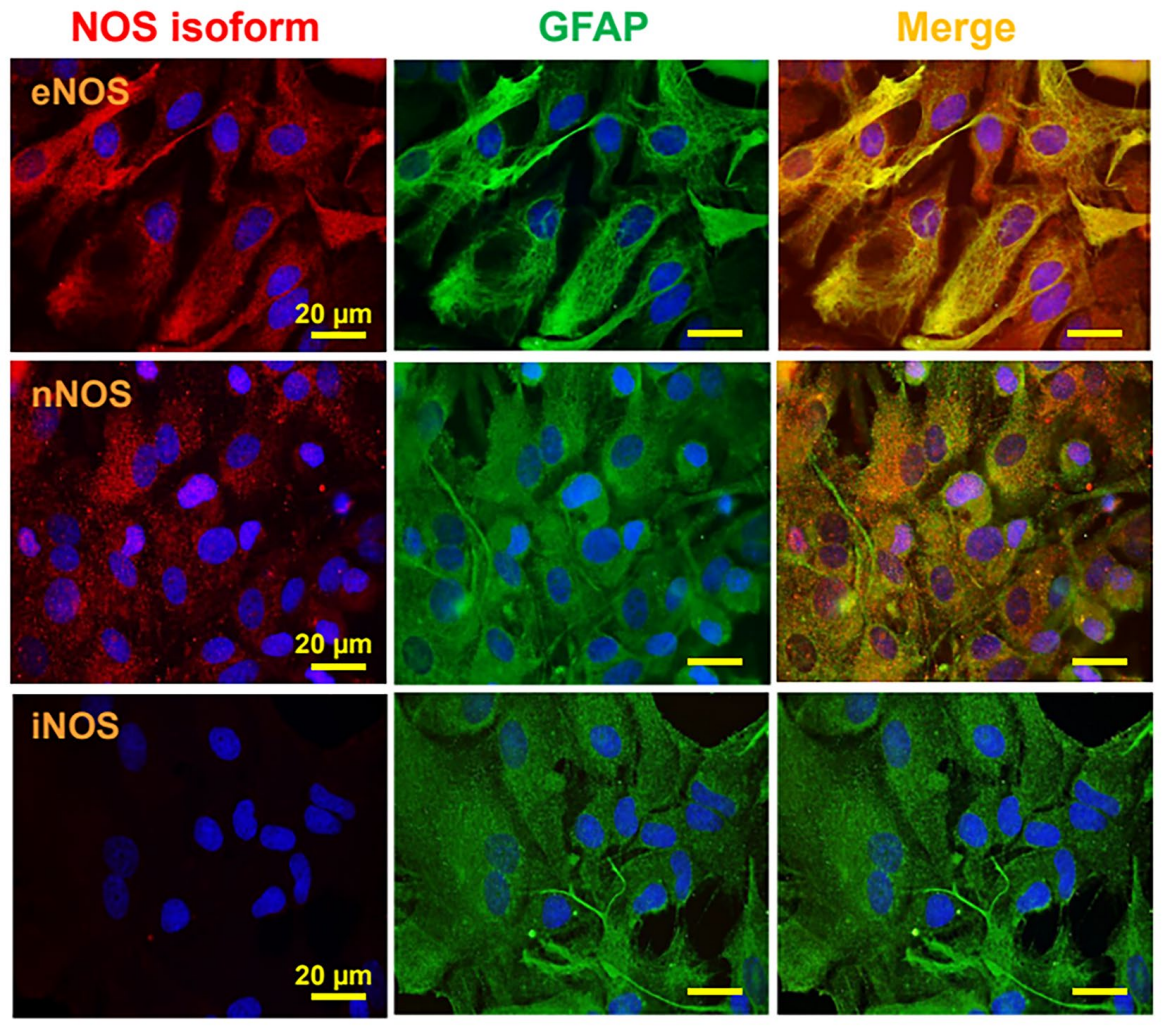
The nitric oxide synthase (NOS) isoforms endothelial (eNOS) and neuronal (nNOS), but not the inducible (iNOS), are found in astrocytes. Detection of the expression of eNOS, nNOS and iNOS (red) by co-immunofluorescence analysis with glial fibrillary acidic protein (GFAP, green) in primary cultures of astrocytes. The cell nuclei are highlighted by the staining with DAPI (blue)

The participation of NO in the regulation of the astrocytic Ca^2+^ signaling was evaluated using N^ω^-nitro-L-arginine (L-NA), a general NOS blocker. Surprisingly, treatment with 100 µM L-NA for 40 min abolished the increase in [Ca^2+^]_i_ activated by glutamate or t-ACPD (Fig. 1A and B). NO signaling is typically mediated by the activation of the cGMP/PKG pathway through the soluble guanylate cyclase. However, inhibition of cGMP production with 10 µM 1H-[1, 2, 4] oxadiazolo[4,3-a]quinoxalin-1-one (ODQ, 15 min application) did not affect the glutamate-activated Ca^2+^ signaling (Fig. 1C), suggesting the involvement of an alternative NO-mediated signaling pathway, such as protein S-nitrosylation, which has emerged as an important “non-classical” mechanism of NO signaling. Consistent with this hypothesis, treatment with 50 µM ascorbic acid, a reducer that can denitrosylate proteins [64], resulted in a similar inhibition of the astrocytic Ca^2+^ signaling to that observed in the presence of L-NA (Fig. 1C) and the response elicited by glutamate or t-ACPD was associated with a clear increase in the global level of astrocytic protein S-nitrosylation, as demonstrated by the immunofluorescence analysis using an anti-S-Nitroso-Cysteine (anti-S-NO) antibody (Fig. 2). Furthermore, the expression of both eNOS and nNOS in primary cultures of astrocytes was also confirmed by immunofluorescence analysis and Western blot. In contrast, the presence of iNOS was not apparent in these cells (Fig. 3 and, Additional file 1: Fig. S1).

### The response to glutamate is associated with Cx hemichannel and Panx-1 channel opening

**Fig. 4:**
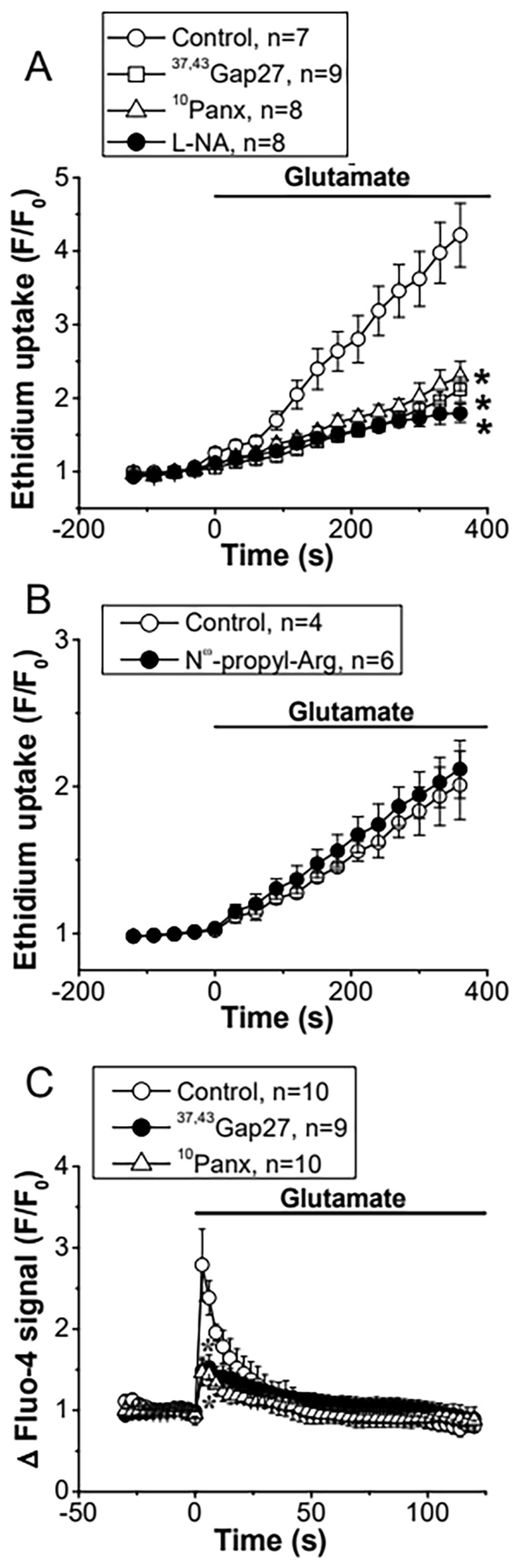
The glutamate-initiated Ca^2+^ signaling in astrocytes is mediated by the opening of Cx hemichannels and Panx-1 channels. **A** Time course of the increase in ethidium uptake observed in primary cultures of astrocytes in response to 10 µM glutamate in control conditions and in the presence of the mimetic peptides ^37,43^Gap27 (100 µM) or ^10^Panx (100 µM) or the NOS inhibitor N^ω^-nitro-L-arginine (L-NA, 100 µM). The peptide ^37,43^Gap27 is a blocker of hemichannels formed by Cx37 or Cx43 and ^10^Panx is an inhibitor of the channels formed by Panx-1. **B** Time course of the increment in ethidium uptake attained before and after the treatment with 60 nM N^ω^-Propyl-L-Arginine (N^ω^-Propyl-L-Arg), a selective inhibitor of the neuronal nitric oxide synthase isoform. Horizontal bars indicate the period of stimulation. **C** Time course of the increase in [Ca^2+^]_i_ induced by glutamate in control conditions and in the presence of ^37,43^Gap27 or ^10^Panx. Values are means ± SEM. *P < 0.05 vs Control by one-way ANOVA plus Bonferroni post hoc test

As NO-mediated S-nitrosylation has been reported to control the function of Cx hemichannels and Panx-1 channels, we evaluated the participation of these channels in the astrocytic Ca^2+^ signaling by assessing ethidium uptake. In line with the activation of this signaling pathway, glutamate activated an increase in ethidium uptake that was strongly inhibited by the treatment for 15 min with the Cx blocking peptide ^37,43^Gap27 (100 µM) or the Panx-1 channel blocker ^10^Panx (100 µM, Fig. 4A and Additional file 1: Fig. S2). It should be noted that application of Cx blocking peptides for short periods of time (< 45 min) only inhibits hemichannel activity, without affecting gap junction channels, which are blocked by longer treatment with these peptides (> 1 h) [65, 66]. Interestingly, the increment in ethidium uptake was also blocked by L-NA (Fig. 4A and Additional file 1: Fig. S2), but not by 40 min application of 60 nM N^ω^-propyl-L-arginine (Fig. 4B and Additional file 1: Fig. S2), a selective inhibitor of nNOS, which, in conjunction, suggest that the response to glutamate is associated with the opening of Cx hemichannels and Panx-1 channels through a mechanism triggered by eNOS-mediated NO production. In addition to ethidium uptake, treatment with ^37,43^Gap27 or ^10^Panx attenuated the glutamate-evoked increase in [Ca^2+^]_i_ (Fig. 4C), confirming the relevance of the Cx and Panx-1-dependent signaling pathway initiated by eNOS in the response to glutamate.

**Fig. 5:**
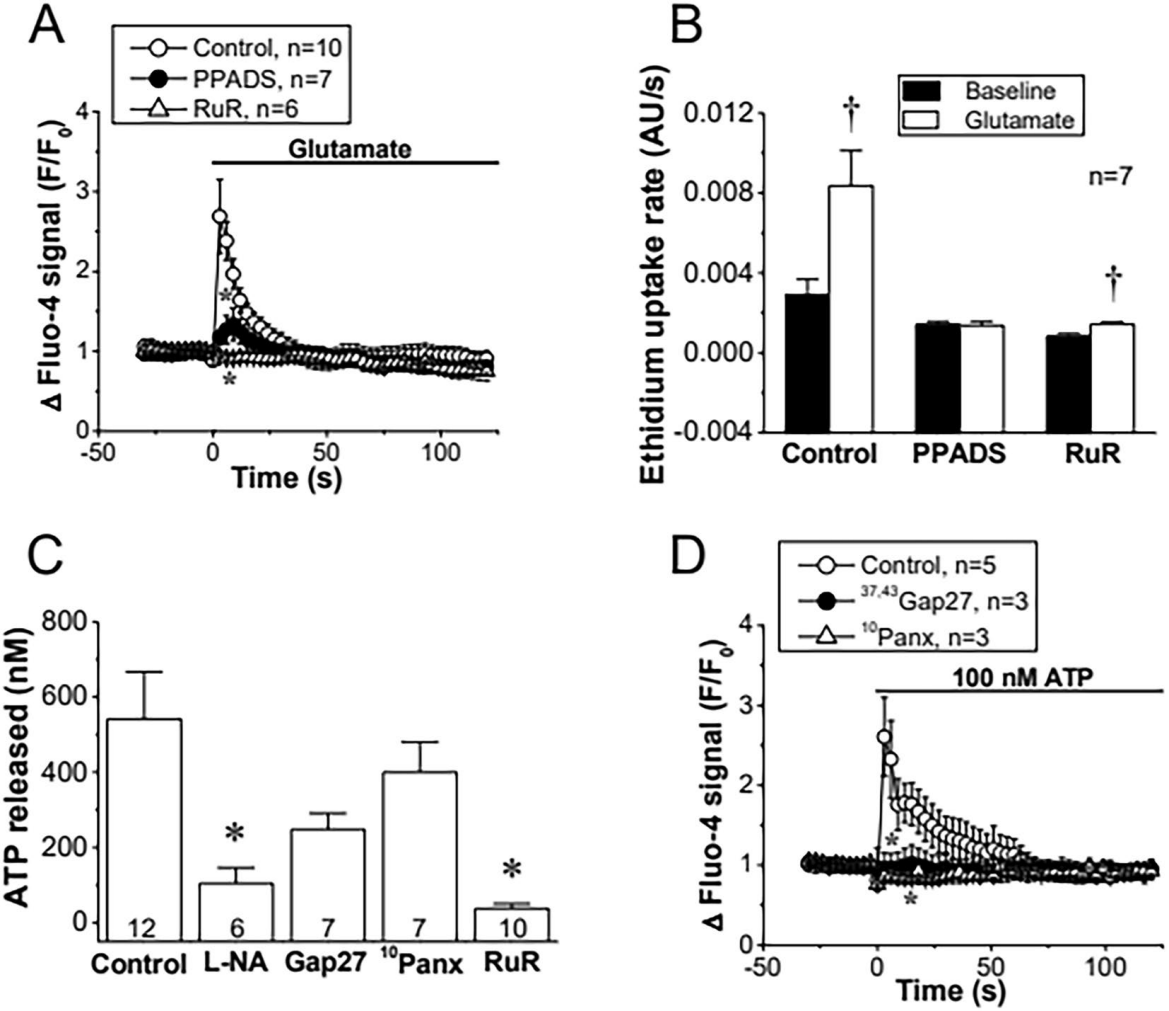
The Cx hemichannel- and Panx-1 channel-mediated Ca^2+^ signaling depends on the activation of purinergic receptors. **A** Time course of the increase in [Ca^2+^]_i_ induced in primary cultures of astrocytes by 10 µM glutamate in control conditions and after the blockade of purinergic receptors with 100 µM PPADS or the inhibition of CALHM1 channels with 20 µM ruthenium red (RuR). **B** Analysis of the increase in ethidium uptake rate induced by glutamate in control conditions and in the presence of PPADS or RuR. The rate of ethidium uptake was assessed by calculating the slope of the increase in fluorescence intensity (expressed as arbitrary units, AU) along the time in basal conditions and during the stimulation with glutamate. **C** ATP release evoked by glutamate in control conditions and after the treatment with 100 µM N^ω^-nitro-L-arginine (L-NA) to inhibit NO production, the mimetic peptide ^37,43^Gap27 (Gap27, 100 µM) to block hemichannels formed by Cx37 or Cx43, ^10^Panx (100 µM) to block Panx1 channels or RuR. ATP was measured after 3 min of stimulation with glutamate. Numbers inside the bars indicate the n value. **D** Time course of the increase in [Ca^2+^]_i_ elicited by 100 nM ATP in primary cultures of astrocytes in control conditions and in the presence of ^37,43^Gap27 or ^10^Panx. Values are means ± SEM. *P < 0.05 vs Control by one-way ANOVA plus Bonferroni post hoc test. ^†^P < 0.05 vs Baseline by paired Student’s t-test

Cx hemichannels and Panx-1 channels can contribute to the astrocytic Ca^2+^ signal by providing a pathway for ATP release and the subsequent purinergic receptor activation. However, blockade of purinergic receptors by the treatment with 100 µM Pyridoxalphosphate-6-azophenyl-2′,4′-disulfonic acid (PPADS) for 15 min not only inhibited the glutamate-elicited Ca^2+^ signaling (Fig. 5A), but also abolished the associated increase in ethidium uptake (Fig. 5B), which indicates that Cx hemichannel and Panx-1 channel activation is found downstream of ATP release. Consistent with this notion, ^37,43^Gap27 or ^10^Panx application did not result in a significant reduction of the ATP release evoked by glutamate (Fig. 5C), but, in contrast, these peptides abolished the increase in [Ca^2+^]_i_ elicited by direct ATP application (Fig. 5D) or the ATP-mediated component of the propagation to neighboring cells of the Ca^2+^ signaling activated by single cell mechanical stimulation (Additional file 1: Fig. S3). It is important to note; however, that L-NA clearly blocked the glutamate- or t-ACPD-evoked ATP release (Fig. 5C and Additional file 1: Fig. S4). Taken together, these results suggest that glutamate triggers the activation of an alternative NO-dependent mechanism of ATP release that leads to Cx hemichannel and Panx-1 channel opening through the stimulation of a purinergic receptor-mediated pathway, which provides a critical contribution to the Ca^2+^ signaling initiated by glutamate.

### Glutamate-evoked Ca^2+^ signaling depends on CALHM1 channel-mediated ATP release

**Fig. 6:**
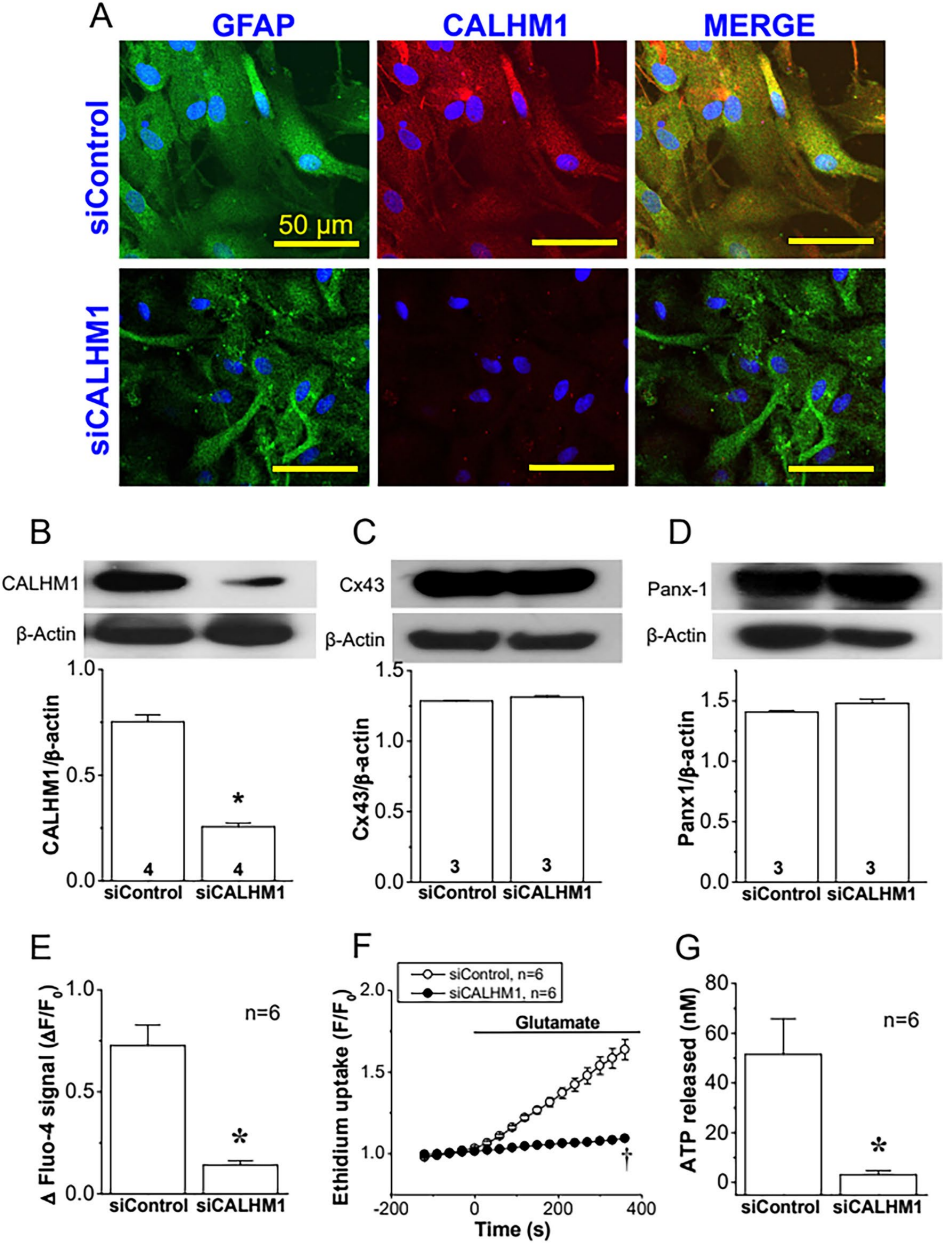
The increase in [Ca^2+^]_i_, activation of Cx hemichannels and Panx-1 channels and ATP release induced by glutamate depends on the expression of CALHM1 channels. **A** Detection of the expression of CALMH1 (red) by co-immunofluorescence analysis with glial fibrillary acidic protein (GFAP, green) in primary cultures of astrocytes treated with a control siRNA (siControl) or a siRNA directed against Calhm1 (siCALHM1). The cell nuclei are highlighted by the staining with DAPI (blue). **B**–**D** Representative Western blot and densitometric analysis of CALHM1 (**B**), Cx43 (**C**) and Panx-1 (**D**) expression in the primary cultures of astrocytes shown in **A**. Numbers inside the bars indicate the n value. The whole images of the representative Western blots are shown in Additional file 1: Fig. S7. **E**, Maximal increase in [Ca^2+^]_i_ observed in response to 10 µM glutamate in astrocytes cultures treated with siControl or siCALHM1. **F** Time course of the increase in ethidium uptake induced by 10 µM glutamate in primary cultures of astrocytes treated with siControl or siCALHM1. The horizontal bar indicates the stimulation period. **G** ATP release attained 3 min after the stimulation with glutamate in the same conditions than those shown in **E** and **F**. Values are means ± SEM. *P < 0.05 vs siControl by unpaired Student’s t-test. ^†^P < 0.05 vs siControl by two-way ANOVA

CALHM1 channels have emerged as an efficient pathway for ATP release, which prompted us to evaluate the participation of these channels in the response to glutamate. In agreement with the participation of these channels, the increase in [Ca^2+^]_i_, ethidium uptake, and ATP release attained in response to glutamate was abolished in the presence of Ruthenium Red (RuR, 20 µM for 15 min), a blocker of CALHM1 channels (Fig. 5A–C). In addition, the expression of CALHM1 was confirmed by Western blot and immunofluorescence analyses in primary cultures of astrocytes and in rat brain sections (Additional file 1: Figs. S5–S7). However, we note that RuR can also affect the activity of TRPV channels, Piezo channels, and Ryanodine receptors (RyR) in addition to CALHM1 channels [67–69]. Therefore, to confirm the participation of CALHM1 in the response to glutamate, primary cultures of astrocytes were treated with a small interference RNA (siRNA) designed to selectively inhibit the expression of CALHM1 (siCALHM1) or a scrambled siRNA, as control (siControl). As expected, the treatment with siCALHM1 resulted in a drastic reduction of CALHM1 signal observed by immunofluorescence staining (Fig. 6A) as well as by Western blot analysis (Fig. 6B and Additional file 1: Fig. S7). In addition, the inhibition of CALHM1 expression was also associated with a strong reduction in the increase of [Ca^2+^]_i_, ethidium uptake, and ATP release (Fig. 6E, F and G), similar to that observed with the application of RuR (Fig. 5). It should be noted that the siCALHM1 treatment did not affect the expression of Cx43 or Panx-1 (Fig. 6C, D and, Additional file 1: Fig. S7).

**Fig. 7:**
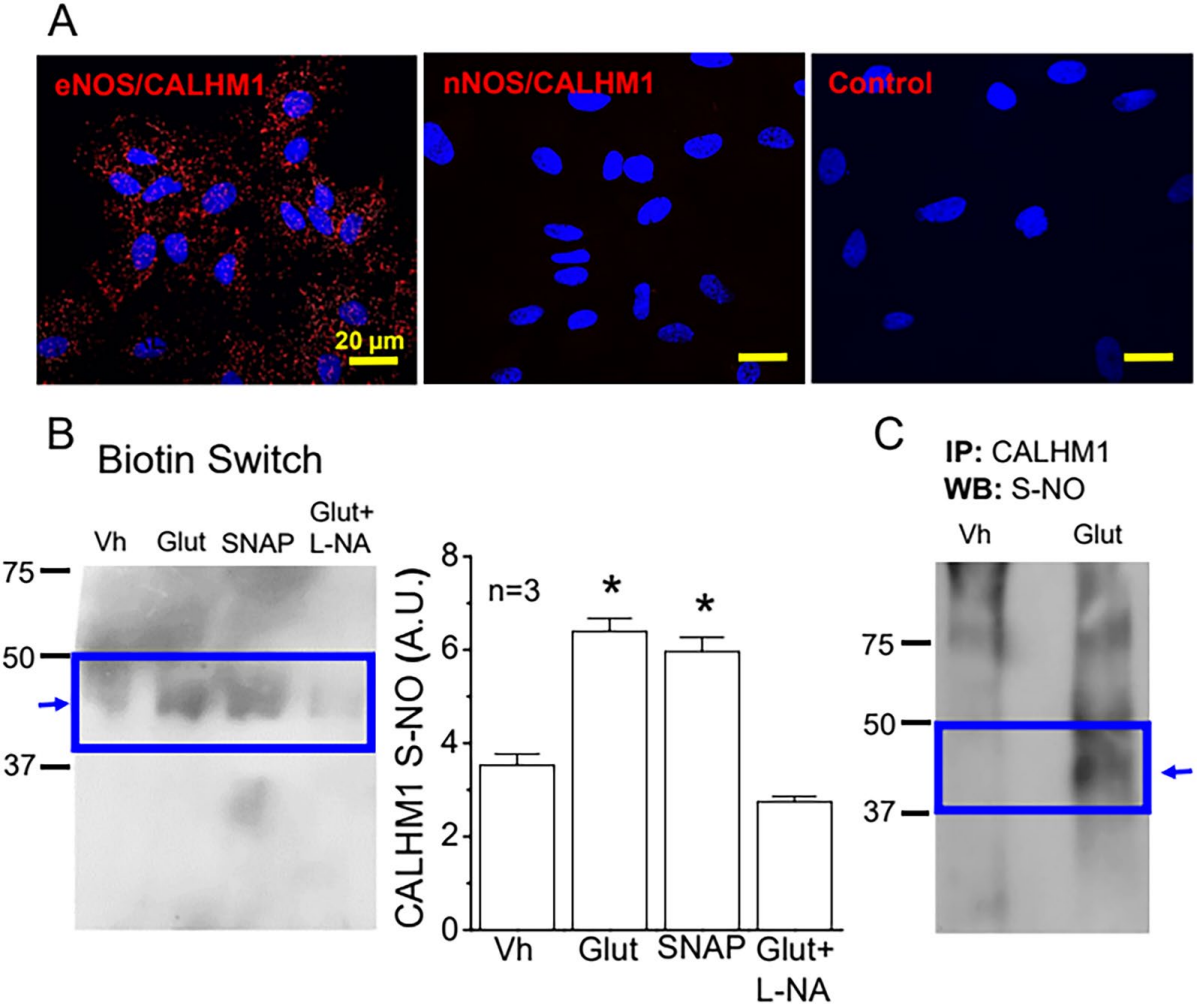
Stimulation with glutamate is associated with the S-nitrosylation of CALHM1 in primary cultures of astrocytes. **A** Analysis performed through Proximity Ligation Assay (PLA) of the spatial association of eNOS or nNOS with CALHM1. Note that CALHM1 is associated with eNOS, but not with nNOS. A control in which primary antibodies were omitted (negative control) is also shown. **B** Representative Western blots (left) and densitometric analysis (right) of the changes in the levels of CALHM1 S-nitrosylation (CALHM1 S-NO) detected by biotin switch in primary cultures of astrocytes 3 min after the stimulation with 10 µM glutamate (Glut), 3 µM SNAP or the vehicle of glutamate (Vh). In addition, the effect of the inhibition of NO production with 100 µM N^ω^-nitro-L-arginine (L-NA) on the glutamate-elicited increase in CALHM1 S-NO is also shown. Variations in the level of CALHM1 S-NO are expressed in arbitrary units (A.U.). **C** Representative Western blot (WB) of CALHM1 S-nitrosylation using an anti-S-Nitroso-Cysteine antibody in astrocytes samples previously submitted to CALHM1 immunoprecipitation (IP). *P < 0.05 vs Vehicle by unpaired Student’s t-test

In combination, these results indicate that the response evoked by glutamate relies on the activation of a NO-initiated, ATP-dependent signaling pathway that appears to be mediated by S-nitrosylation. Therefore, to evaluate the possible functional regulation of CALHM1 by NO production, we assessed the spatial relation between this protein with eNOS and nNOS by Proximity Ligation Assay (PLA) and the direct CALHM1 S-nitrosylation by Biotin Switch and immunoprecipitation. Interestingly, in line with the divergent effect of L-NA and N^ω^-propyl-L-arginine on the activation of Cx hemichannels and Panx-1 channels (Fig. 4), the analysis of PLA revealed that CALHM1 is found in close spatial proximity with eNOS, but not with nNOS (Fig. 7A). As expected, PLA signal was negative in control experiments in which primary antibodies were omitted (Fig. 7A). In addition to the association with eNOS, the Biotin Switch analysis showed that stimulation with glutamate leads to an increase in the level of S-nitrosylation of CALHM1 (Fig. 7B), which was confirmed by Western blot detection of S-nitrosylated cysteine residues in astrocyte samples subjected to CALHM1 immunoprecipitation (Fig. 7C). The glutamate-evoked increase in CALHM1 S-nitrosylation was prevented by the blockade of NO production with L-NA (Fig. 7B). Then, these results indicate that NO-mediated S-nitrosylation is a critical event of the response activated by glutamate and, consistent with this notion, stimulation with 3 µM S-nitroso acetyl penicillamine (SNAP), a NO donor, evoked an increase in CALHM1 S-nitrosylation similar to that observed in response to glutamate (Fig. 7B) and a RuR-sensitive Ca^2+^ signaling (Additional file 1: Fig. S8).

### CALHM1 channel opening is enhanced by NO-mediated S-nitrosylation

**Fig. 8:**
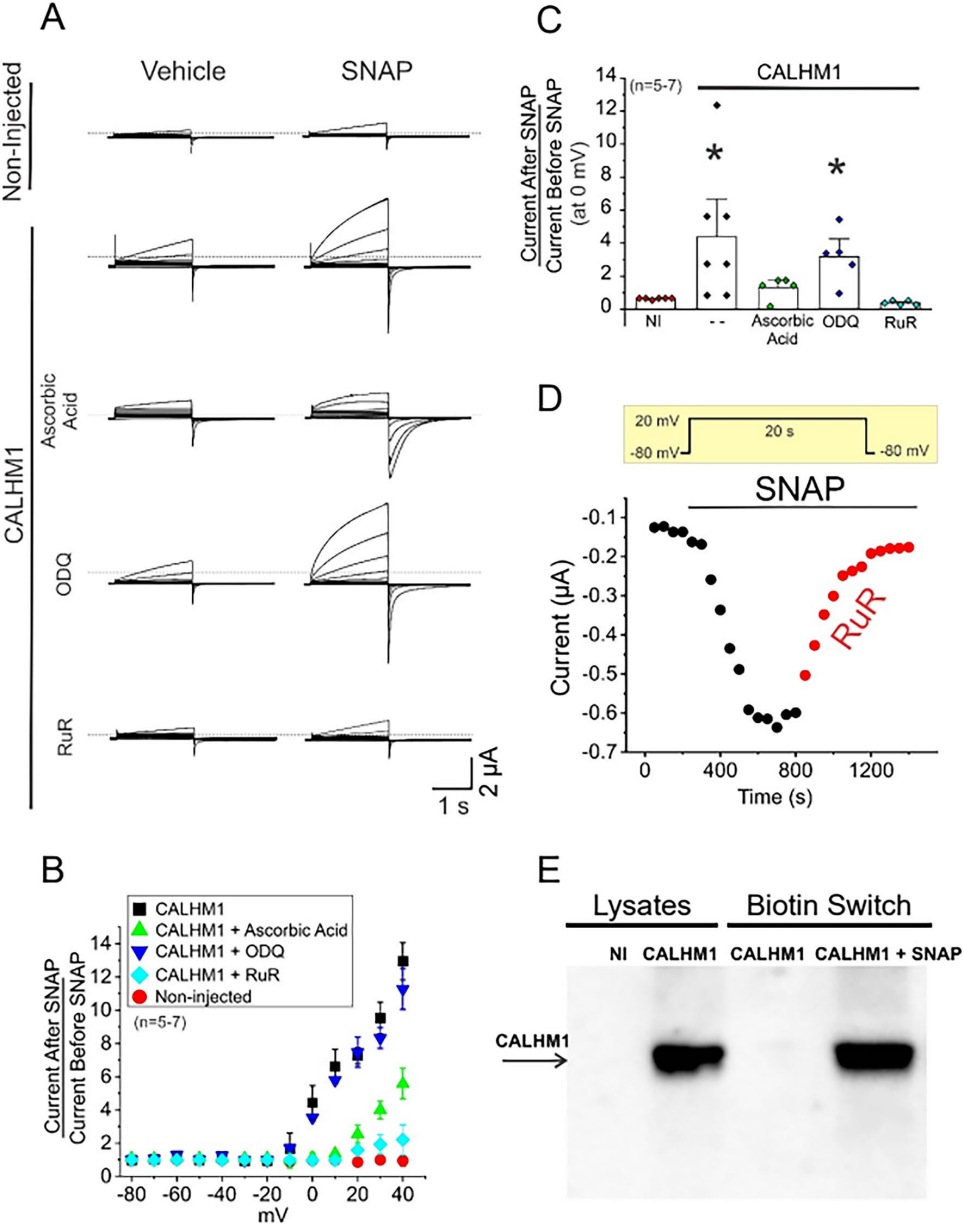
NO activates CALHM1 channels in a heterologous expression system.** A** Representative current traces before and after application of 10 μM SNAP in a non-injected oocytes (NI) or oocytes expressing CALHM1 obtained at 1.8 mM extracellular Ca^2+^. Three CALHM1 expressing oocytes were additionally treated with 50 µM ascorbic acid, 10 µM ODQ or 20 µM ruthenium red (RuR). Oocytes were clamped to − 80 mV, and square pulses from − 80 mV to + 40 mV (in 10 mV steps) were then applied for 2 s. At the end of each pulse, the membrane potential was returned to − 80 mV. Note that ODQ did not affect NO-induced CALHM1 currents. **B** Normalized currents were obtained from the ratio between recorded current after and before 10 µM SNAP treatment. **C** Normalized currents at 0 mV oocytes resting membrane potential before and after 10 µM SNAP stimulation. Comparisons between groups were made using two-way ANOVA plus Tukey post-hoc test, *P < 0.05 vs Non-Injected (NI). **D** Time course of tail current peaks after reaching current saturation during a depolarizing pulses from − 80 to 20 mV (yellow box). Tail current peaks were obtained in the absence and presence of SNAP, and during RuR application. **E** Representative Western blot showing the expression and NO-mediated S-nitrosylation of CALHM1 in oocytes. From left to right, the first two lanes correspond to Western blots in non-injected oocytes (NI) and oocytes expressing CALHM1, and the next two lanes show the Biotin Switch analysis of CALHM1 S-nitrosylation observed in oocytes expressing CALHM1 in control conditions (lane 3) and after the stimulation with SNAP (lane 4)

To directly demonstrate that NO activates CALHM1 channels, we assessed the effect of SNAP on CALHM1 currents using the two-electrode voltage-clamp technique. In oocytes expressing CALHM1 channels, application of 10 µM SNAP induced an increase in CALHM1-mediated currents observed at positive membrane potentials (Fig. 8A and B). In contrast, plasma membrane conductance was not affected by 10 µM SNAP in control oocytes (non-injected) at all tested voltages (Fig. 8A and B). In CALHM1 expressing oocytes, SNAP application evoked a fourfold increase in the currents attained at 0 mV (Fig. 8C). Consistent with the involvement of NO-mediated S-nitrosylation in the SNAP-induced CALHM1 current potentiation, the response was not sensitive to the inhibition of soluble guanylate cyclase with 10 µM ODQ, but, in contrast, it was strongly reduced by the treatment with ascorbic acid (Fig. 8A–C). In addition, 20 µM RuR abolished the NO-induced CALHM1 current potentiation (Fig. 8A–C).

Next, we analyzed the time course of the CALHM1 currents observed in response to SNAP during a depolarizing pulse from − 80 to 20 mV. Figure 8D shows a representative trace of the rate of current change before and after NO donor application in an oocyte expressing CALHM1 channels. Stimulation with NO, after the stabilization of the current at 200 s, elicited an increase in the inward current that reached a maximum at ~ 800 s and further application of 20 µM RuR resulted in a gradual reduction in the inward current, which returned to the initial values observed previous SNAP application. Overall, these results suggest that NO activates CALHM1 channels by S-nitrosylation, which was biochemically confirmed by the biotin switch assay, since S-nitrosylated CALHM1 was only detected in oocytes expressing CALHM1 proteins that were stimulated with 10 µM SNAP (Fig. 8E).

## Discussion

Astrocytes have emerged as a key cellular pathway in the modulation of the intercellular communication network in the brain and intracellular Ca^2+^ is an essential signaling for astrocyte function [7, 70]. It is thought that ATP release via Cx43 hemichannels and Panx-1 channels is involved in the control and coordination of astrocyte-mediated Ca^2+^ signaling [36, 42]. However, in the present work, we found that glutamate-induced Ca^2+^ signaling depended on previously unrecognized players in rat astrocytes in vitro. Our results indicate that astrocytic ATP release attained in response to glutamate is mainly mediated by CALHM1 channels. The mechanism involved in this response is initiated by mGluR-depended NO signaling, which leads to CALHM1 channel opening by S-nitrosylation and the subsequent ATP release through these channels. The subsequent purinergic signaling triggered by ATP release amplifies the initial mGluR-mediated astrocytic Ca^2+^ signal via the activation of Cx43 hemichannels and Panx-1 channels.

NO is a very versatile signaling molecule that plays critical roles in both physiological and pathophysiological conditions [71, 72]. In the CNS, NO is an important regulator of brain function that can be released by pre- or post-synaptic endings to work as anterograde or retrograde neurotransmitter [73]. However, in addition to neurons, the expression of different isoforms of NO-synthetizing enzymes has also been observed in astrocytes in physiological conditions [57, 61]. Although NO signaling in these glial cells has not attracted much attention, in the present study, we demonstrated that inhibition of NO production prevented the increase in astrocytic [Ca^2+^]_i_ activated by the stimulation of mGluR with glutamate or t-ACPD (Fig. 1), which indicates that NO plays a central role in the control of astrocyte function. The response to NO production is classically mediated by the activation of soluble guanylate cyclase and the cGMP/PKG pathway [74], but, interestingly, blockade of cGMP production did not affect the glutamate-induced astrocyte Ca^2+^ signaling (Fig. 1), suggesting the involvement of an alternative mechanism of NO signaling, such as NO-dependent S-nitrosylation. Consistent with this hypothesis, the response to glutamate and t-ACPD was associated with a striking increase in global protein S-nitrosylation (Fig. 2) that was fully prevented by the treatment with L-NA. It is important to note that we confirmed the presence of eNOS and nNOS in primary cultures of brain cortex astrocytes (Fig. 3). As expected, the expression of iNOS was undetectable, which is in line with previous reports showing that the expression level of this NOS isoform is very low in physiological conditions and is upregulated in activated astrocytes [62].

Activation of eNOS or nNOS depends on an increase in [Ca^2+^]_i_ and NO production by astrocytes has been observed to participate in the control of astrocytic spontaneous Ca^2+^ transients [75] and in the astrocytic ATP release [76]. Although the increase in [Ca^2+^]_i_ triggered by the stimulation of mGluR in astrocytes is initiated by the release of intracellular Ca^2+^ from the endoplasmic reticulum store, the contribution to the response of purinergic P2 receptor activation through astrocytic ATP release has also been recognized [77]. In this context, Cx hemichannels and Panx-1 channels have been found to provide an important pathway for ATP release in astrocytes [36, 42]. Cx30 and Cx43 are the most prominent Cx proteins expressed in astrocytes and, in these cells, NO-mediated S-nitrosylation has been reported to control the opening of Cx43-formed hemichannels [32]. In addition, NO has also been shown to affect the Panx-1 channel function through the cGMP pathway or directly by S-nitrosylation [55, 56]. In agreement with these observations, we confirmed the activation of Cx43 hemichannels and Panx-1 channels in the glutamate-initiated astrocytic Ca^2+^ signaling by a NO-dependent pathway (Fig. 4). However, the opening of Cx43 hemichannels or Panx-1 channels did not provide the main pathway for ATP release in response to glutamate and, in contrast to this notion, the opening of these channels was a consequence of the purinergic receptor activation, most likely, by ATP (Fig. 5). Taken together, these results indicate that the mGluR signaling is coupled to the activation of an alternative NO-dependent mechanism of ATP release that, subsequently, leads to the opening of Cx43 hemichannels and Panx-1 channels through the stimulation of a purinergic receptor-mediated pathway. It is interesting to note that although both isoforms, eNOS and nNOS, were detected in the primary cultures of astrocytes (Fig. 3), the NO production that triggered the ATP-mediated signaling cascade can mostly be attributed to the specific activation of eNOS, because the opening of Cx43 hemichannels and Panx-1 channels was prevented by the general NOS blocker L-NA, but not by selective nNOS inhibition with N^ω^-propyl-L-arginine (Fig. 4).

As Cx43 hemichannels and Panx-1 channels did not account for the ATP release observed in response to glutamate, we analyzed the potential participation of CALHM1 channels in the astrocytic ATP signaling. CALHM1 is a plasma membrane protein that exhibit a similar topology of Cxs and forms channels with a pore functional diameter of ~ 1.42 nm at its narrowest region [78, 79]. Although these channels were initially thought to be formed by the assembly of six CALHM1 monomers, recently, Cryo-electron microscopy analyses have led to propose that they present an octameric architecture [80]. CALHM1 channels were first involved in the regulation of Ca^2+^ signaling in the brain and, in this context, it was suggested that they participate in the control of neuronal excitability and in the long-term synaptic potentiation [81, 82]. Nevertheless, more recently, the attention has been focused on the ATP-releasing function of these channels that has been observed in several tissues [48]. Although the relevance of CALHM1 as a pathway for ATP release has not been determined in the brain, our data strongly suggest that CALHM1 channels mediate the transmembrane ATP signaling triggered by glutamate in astrocytes. In line with this notion, treatment of primary cultures of astrocytes with RuR, a nonspecific CALHM1 blocker, or with a siRNA designed to downregulate the CALHM1 expression not only prevented the glutamate-induced ATP release, but also abolished the increase in [Ca^2+^]_i_ and the Cx43 hemichannel- and Panx-1 channel-mediated ethidium uptake observed in response to mGluR stimulation (Figs. 5 and 6). It is important to note that these results are not only supported by the demonstration that the siRNA-induced downregulation of CALHM1 did not affect the expression of Cx43 or Panx-1 (Fig. 6), but also by the detection of CALHM1 expression in astrocytes in both primary cultures and intact brain sections (Fig. 6 and Additional file 1: Fig. S5, S6). In this context, we note that the immunofluorescence analysis shows that CALHM1 is found in plasma membrane, as expected, but also in intracellular compartments, which is consistent with the cellular distribution of this protein observed previously, where the expression of CALHM1 was strongly detected in endoplasmic reticulum and plasma membrane [83, 84]. Taken together, these results indicate that astrocytes express CALHM1 and the channels formed by this protein provide a critical pathway for the ATP release-triggered signaling initiated by the activation of mGluR, which is very likely to play a central role in the astrocyte-mediated modulation of synaptic activity.

In addition to provide the main transmembrane pathway for ATP release in the response of astrocytes to glutamate, CALHM1 channels are also permeable to Ca^2+^ [78, 79], and therefore, in parallel to trigger the activation of the ATP release-mediated signaling, these channels might participate in the initial increase in [Ca^2+^]_i_. In this line and consistent with a direct contribution of a Ca^2+^ influx through CALHM1 channels, blockade of the activity (RuR) or the expression (siRNA-mediated downregulation) of these channels completely abolished the glutamate-activated Ca^2+^ signaling, whereas a component of the response prevailed after inhibiting the ATP-dependent downstream signaling events with PPADS or the blocking peptides ^37,43^Gap27 and ^10^Panx (Figs. 4 and 5). Interestingly, the magnitude of the reduction in the Ca^2+^ signaling attained with the inhibition of purinergic receptors, Cx43 hemichannels or Panx-1 channels was not statistically different (Additional file 1: Fig. S9) and the increment in [Ca^2+^]_i_ observed in response to ATP application was prevented by ^37,43^Gap27 and ^10^Panx (Fig. 5), which confirms that Cx hemichannel and Panx-1 channel opening is downstream of ATP release and suggests that the ATP-induced Ca^2+^ signaling is mostly dependent on the activation of these channels. Interestingly, astrocytes are functionally coupled and, in addition to gap junctions, ATP release provides an important pathway for the propagation of Ca^2+^ signals through neighboring astrocytes by the activation of Cx43 hemichannels and Panx-1 channels (Additional file 1: Fig. S3), although the mechanisms involved in this response require further investigation.

The increase in astrocytic [Ca^2+^]_i_ evoked by glutamate depended on the activation of a cGMP-independent, NO-mediated signaling and the response initiated by mGluR stimulation was associated with a strong increment in total protein S-nitrosylation (Figs. 1 and 2), suggesting the involvement of this posttranslational modification in the response. In contrast to the NO-initiated cGMP pathway, the activation of the S-nitrosylation signaling mechanism is preferentially observed close to the NO source, where NO concentration is higher [85]. In this context, it is interesting to note that the PLA analysis revealed that CALHM1 is found in close spatial association with eNOS, but not with nNOS (Fig. 7), which further supports the involvement of the endothelial isoform of the enzyme in the activation of the CALHM1 channel-mediated mechanism that controls the glutamate-elicited Ca^2+^ signaling in astrocytes. Interestingly, the association of CALHM1 with eNOS was observed in both in the intracellular compartment and in the plasma membrane, which suggest that eNOS may also regulate the trafficking of CALHM1. In addition, consistent with the notion that the interaction between CALHM1 and eNOS is functional, our results indicate that the response activated by glutamate is associated with a notable increase in CALHM1 S-nitrosylation (Fig. 7), which supports the proposal that CALHM1 channels are directly activated by NO-mediated S-nitrosylation of this protein. To confirm this hypothesis, we directly analyzed the effect of NO on the activity of CALHM1 channels expressed in oocytes. Consistent with the NO-dependent opening of CALHM1 channels observed in primary cultures of astrocytes, activation of these channels by depolarizing voltage steps was dramatically augmented in the presence of NO, which was also associated with CALHM1 S-nitrosylation (Fig. 8).

## Conclusion

**Fig. 9:**
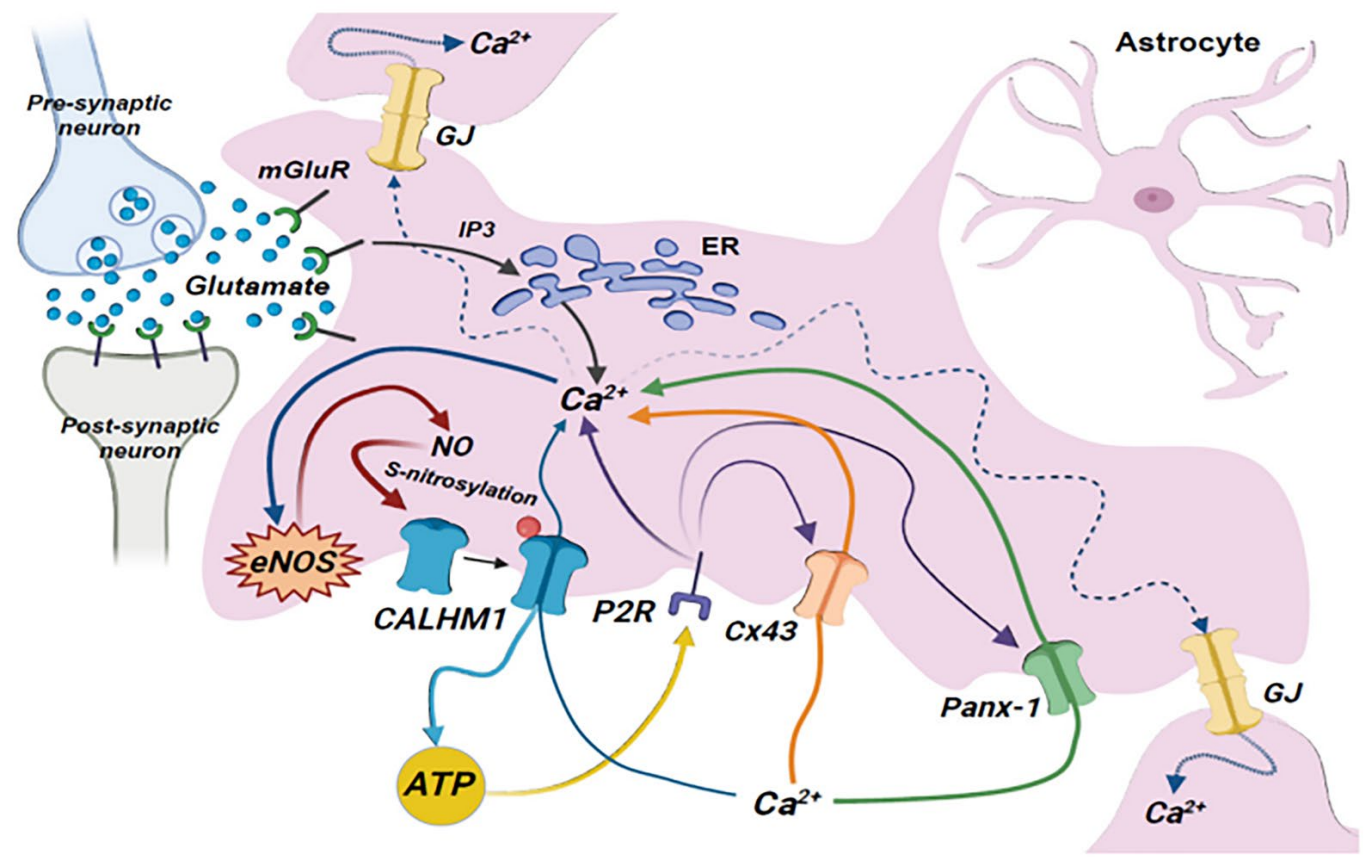
Schematic model of the signaling events that mediate the increase in [Ca^2+^]_i_ initiated by metabotropic glutamate receptor (mGluR) activation in astrocytes. Glutamate released during an increase in neuronal activity can exit the synaptic cleft and activate receptors on astrocyte processes. The activation of astrocyte mGluRs leads to an initial increase in [Ca^2+^]_i_ by the release of Ca^2+^ from the intracellular Ca^2+^ stores through activation of an inositol (1,4,5)-triphosphate (IP_3_)-mediated pathway. This astrocytic Ca^2+^ signal triggers an increment in nitric oxide (NO) production by the endothelial NO synthase isoform (eNOS), which, in turn, evokes the opening of CALHM1 channels by the S-nitrosylation of this protein. The activation of CALHM1 channels play a pivotal role in the response by providing a pathway for ATP release and the sequential activation of P2 receptors (P2R), leading to the opening of Cx43 hemichannels and Panx-1 channels. Finally, the Ca^2+^ influx through these membrane channels contributes to amplify the intracellular Ca^2+^ store-initiated Ca^2+^ signaling. In addition, the increase in [Ca^2+^]_i_ can be coordinated through the propagation of an inter-astrocyte Ca^2+^ signal via ATP release or directly by gap junction communication (GJ)

Our data indicate that astrocytes express functional CALHM1 channels, and the presence of these channels plays a critical role in a complex signaling pathway that leads to the generation of the astrocytic Ca^2+^ signaling triggered by glutamate. Activation of mGluR is coupled to an increase in eNOS-mediated NO production, which, in turn, elicits the S-nitrosylation of CALHM1, with the consequent activation of the channels formed by this protein. Finally, CALHM1 channel opening is essential to amplify the intracellular Ca^2+^ store-initiated Ca^2+^ signaling by providing a pathway for ATP release and the sequential activation of P2 receptors, Cx43 hemichannels and Panx-1 channels (Fig. 9). Changes in [Ca^2+^]_i_ account for astrocyte excitability; therefore, the findings of the present work suggest that the NO/CALHM1 channel signaling pathway plays a key role in astrocyte function.

## Methods

Neonatal Sprague–Dawley rats of 1-to-2-day-old were obtained from the Research Animal Facility of the Pontificia Universidad Católica de Chile. All studies were approved by the Institutional Bioethics Committee (protocol ID 170321004) and the experiments were conducted according to the Helsinki Declaration and the Guiding Principles in the Care and Use of Laboratory Animals endorsed by the American Physiological Society.

### Primary cultures of astrocytes

Primary cultures of astrocytes were prepared from brain cortex of 1-to-2-day-old neonatal rats as described previously by Schildge et al. [86]. Briefly, brain cortices were dissected free of meninges and minced in cool phosphate-buffered saline (PBS) and incubated at 37 °C for 30 min in a PBS solution containing 5 mM EDTA and 0.25% Trypsin (Biological Industries, Cromwell, Connecticut, USA Cat. #03–051-5B). After removing the enzyme solution, cells were pelleted and resuspended in DMEM culture medium (Gibco® by Life Technologies™, Carlsbad, California, USA Cat. #31,600–034) supplemented with 3.7 g/L NaHCO_3_, 10% FBS (Gibco® by Life Technologies™, Carlsbad, California, USA Cat. #16,000–044), 100 units/ml penicillin, 100 µg/ml streptomycin and 0.25 µg/ml fungizone (Gibco® by Life Technologies™, Carlsbad, California, USA, Cat. #15,240). Thus, cells were plated in T75 Nunc flask and grown for ∼ 1 week at 37 °C in a 5% CO_2_─95% air atmosphere at nearly 100% relative humidity to reach a confluence of ~ 80% and the cell culture was shaken (200 rpm) for 20 h at 37 °C to separate astrocytes from neurons, microglia and oligodendrocytes, as demonstrated by Schildge et al. [86]. The purity of astrocyte cultures was confirmed by immunofluorescence analysis (Additional file 1: Fig. S10). Astrocytes were seeded onto sterile glass coverslips and the experiments were performed after 2 or 3 days in astrocyte cultures with a confluence of 70 to 80% in which the culture media was replaced by a MOPS-buffered Tyrode saline solution (in mM: NaCl 118; KCl 5.4; CaCl_2_ 2.5; KH_2_PO_4_ 1.2; MgSO_4_ 1.2; MOPS 10; glucose 11, pH 7.4).

### Measurement of changes in intracellular Ca^2+^ concentration

Changes in [Ca^2+^]_i_ were detected using the fluorescent Ca^2+^ indicator, Fluo 4 (Life Technologies™, Carlsbad, California, USA Cat. #F23917). Primary cultures of astrocytes were incubated with 10 µM Fluo 4-AM for 1 h at room temperature (~ 25 °C) to upload the cells with Fluo 4 and changes in [Ca^2+^]_i_ were analyzed after 20 min of equilibration. Fluo 4-AM was dissolved in DMSO and, thus, prepared in MOPS-buffered Tyrode saline solution. The fluorescent signal was recorded by epifluorescence (exciter: 470–490 nm, band pass filter; emission; 515 nm, long pass filter) using an Olympus BX50 WI microscope and an intensified CCD camera (Retiga Fast 1394, Q Imaging).

Image acquisition and data analysis were performed using IPLab software (Scanalytics, Inc). Images of astrocytes were acquired at a frequency of 3 s in time-lapse experiments and the fluorescence intensity was analyzed using the software ImageJ (U. S. National Institutes of Health, Bethesda, Maryland, USA). Variations in Fluo-4 fluorescence of each cell in the microscope field were analyzed and changes in [Ca^2+^]_i_ were expressed as the ratio, F/F_0_, where F is the fluorescence observed during the stimulation period and F_0_ is the baseline fluorescence value. The average of all analyzed cells in an experiment was considered one measurement, and then, the n value indicates the number of independent cell cultures. Hence, no change in Fluo-4 fluorescence corresponds to Fluo-4 ratio=l.

### Activity of connexin hemichannels and pannexin channels

Opening of Panx channels or Cx hemichannels was evaluated by measuring ethidium uptake, as described previously [87]. Primary cultures of astrocytes plated on glass cover slips were incubated in MOPS-buffered Tyrode solution containing 5 µM ethidium bromide and the ethidium fluorescence signal was recorded over time. Fluorescence intensity of ethidium intercalated in nucleic acids was examined by epifluorescence (exciter: 530–550 nm, emission: 590 nm) using an Olympus BX50 WI microscope and intensified CCD camera (Retiga Fast 1394, Q Imaging) controlled by the IP Lab software (Scanalytics, Inc). Images were acquired every 30 s in time-lapse experiments and the fluorescence intensity was analyzed using the software ImageJ (U. S. National Institutes of Health, Bethesda, Maryland, USA). Changes in the fluorescence intensity of each cell in the microscope field were averaged and expressed as the variations of the fluorescence intensity, F/F_0_, where F is the fluorescence observed during the stimulation period and F_0_ is the baseline fluorescence value.

### Electrophysiology

The two-electrode voltage clamp (TEVC) technique and *Xenopus* oocytes were used to evaluate the activation of CALHM1 currents in response to SNAP, a NO donor. Oocytes were enzymatically obtained from ovaries of female *Xenopus laevis* that were purchased from Xenopus Express. The human CALHM1 clone was commercially synthesized by Epoch Life Science and Nhe1-linearized hCALHM1 was transcribed in vitro to cRNAs using the T7 Message Machine kit (Ambion). Electrophysiological data were collected at room temperature (20 °C–22 °C) using pCLAMP10 software (Molecular Devices Corporation). The extracellular recording solutions contained (in mM) 118 NaCl, 2 KCl, and 5 HEPES and 1.8 Ca^2+^ (pH 7.4). Currents from oocytes were recorded 1–2 days after cRNA injection, using a Warner OC-725 amplifier (Warner Instruments), and were sampled at 2 kHz with a low-pass filter at 0.5 kHz. Microelectrode resistances were between 0.1 and 1.2 MΩ when filled with 3 M KCl. CALHM1 currents were assessed in control conditions and 10 min after SNAP application. Oocytes were injected with 120 μM BAPTA before TEVC recordings to prevent the activation of chloride currents by Ca^2+^. We used at least three oocytes per each independent frog.

### ATP measurements

Changes in ATP concentration were measured in the bathing solution of cultured astrocytes using the ATP determination Kit from Molecular Probes, Life technologies (Cat. #A22066, Eugene, OR, USA) and the chemiluminescent signal was detected with a luminometer Turner TD-20e (Turner BioSystems Inc).

### Western Blotting

Proteins of cultured astrocytes were separated by 10% SDS-PAGE and transferred onto a PVDF membrane (Pierce, Rockford, IL). The primary and secondary antibodies (Pierce, Rockford, IL) were incubated using the Signal Enhancer HIKARI (Nacalai Tesque, INC, Japan) and the protein bands were detected with the SuperSignal^®^ West Femto (Pierce, Rockford, IL). Molecular mass was estimated with pre-stained markers (BioRad, Hercules, CA). Protein bands were analyzed using the ImageJ software. To evaluate the changes of protein expression in astrocytes cultures treated with a siRNA designed to target rat *Calhm1* mRNA, blots were developed for of CALHM1, Cx43 or Panx-1, and then, stripped, and re-probed for ß-actin to express the changes as the ratio of the protein over ß-actin.

### Biotin switch assay

Changes in S-nitrosylation levels were analyzed with the Biotin switch assay as described by Jaffrey and Snyder [88]. Briefly, samples of 200 μg protein were treated with S-methyl methanethiosulfonate (MMTS, Sigma Aldrich, USA, Cat. #64,306) for 1 h at 50 °C in the dark to block cysteine-free thiols, and then, proteins were precipitated with ice-cold acetone for 24 h at − 20 °C. The S-nitrosylated cysteine residues were reduced with 2.5 mM sodium ascorbate (Sigma Aldrich, USA, Cat. #A7506) and 4 mM N-[6-(Biotinamido) hexyl]-3′-(2′-pyridyldithio) propionamide (HPDP-biotin, Thermo Scientific, Cat. #21,341) was used to label the reduced thiols with biotin. Samples were incubated for 1 h with agarose-conjugated streptavidin beads (Thermo Scientific, Cat. #20,353) and centrifuged to pull down HPDP-biotinylated proteins, which were finally separated by SDS-PAGE to be detected with specific antibodies.

### Immunoprecipitation

The level of CALHM1 S-nitrosylation was also assessed by immunoprecipitation, as described previously [89]. Astrocyte homogenate samples containing 100 µg of protein were pre-cleared by the treatment with 30 µl Parsobin^®^ (Calbiochem, Cat. #507,861) for 1 h at 4 °C. Samples were thus centrifuged and supernatants were incubated with polyclonal anti-CALHM1 antibody (1 µg antibody: 100 µg protein) for 2 h at 4 °C. Bound protein was precipitated with 30 µl Parsobin^®^ (2 h at 4 °C) and subsequent centrifugation to 5.300 g. The precipitated protein was washed three times with lysis buffer and the pellet was resuspended in Laemmli’s buffer to be submitted to SDS-PAGE and Western blot analysis to detect the S-nitrosylation of the protein using an anti-S-NO antibody (Sigma Aldrich, USA, Cat. #N5411).

### Inhibition of CALHM1 expression

The expression of CALHM1 was downregulated using a siRNA designed to target rat *Calhm1* mRNA that was obtained from QIAGEN (Düsseldorf, Germany, Cat. #SI02901241). Transfections were performed using the HiPerFect transfection kit (QIAGEN, Cat. #301,704) as indicated in the manufacturer’s protocols. Astrocyte cultures at ~ 80% confluence were subjected to transfection for 6 h, and then, transfection medium was replaced with fresh medium supplemented with 2% FBS. CALHM1 expression was evaluated from 48 to 96 h and the maximum reduction in protein expression was found at 72 h.

### Immunofluorescence analysis

Astrocyte monolayers were fixed with 2% paraformaldehyde in PBS, blocked with 0.5% BSA in PBS and incubated with the corresponding rabbit polyclonal primary antibody, mouse monoclonal primary antibody or both in the case of co-immunofluorescence analysis, and then, with the appropriate Alexa-568-labeled goat anti-rabbit or anti-mouse secondary antibody (Invitrogen Molecular Probes, USA, Cat. #A11011 or Cat. #A11004, respectively), or Alexa-488-labeled goat anti-rabbit or anti-mouse secondary antibody (Invitrogen Molecular Probes, USA, Cat. #A11008 or Cat. #A10680, respectively) using the Signal Enhancer HIKARI (Nacalai Tesque, INC, Japan) as indicated by the manufacturer. The fluorescent signal was examined using an Olympus BX41 WI microscope and a CCD camera (Jenoptik ProgRes C5).

### Analysis of protein-to-protein association

The subcellular distribution and possible spatial association of eNOS with Cx43, Panx-1 or CALHM1 was evaluated by PLA (Duolink^®^ II, OLINK Bioscience, Sweden Olink). Primary cultures of astrocytes were fixed, blocked, and incubated with two primary antibodies from different species, which were, then, detected using oligonucleotide-conjugated secondary antibodies (PLA probe anti-mouse PLUS, Cat. #92,001, and PLA probe anti-rabbit MINUS, Cat. #92,005), as described in the manufacturer’s protocols. If the target proteins are closer than 20 nm, the oligonucleotides can be used as template for DNA ligase-mediated joining of additional oligonucleotides to form a circular DNA molecule, which was amplified using hybridizing fluorophore-labeled oligonucleotides. Images were visualized with a Nikon eclipse Ti confocal microscope and the NIS Elements ND2 program.

### Primary antibodies

The following primary antibodies were used in Western blot, Immunofluorescence and PLA analysis: mouse monoclonal anti-Cx43 (BD Transduction Laboratories™, USA, Cat. #610,062), rabbit polyclonal anti-Panx-1 (Sigma-Aldrich, USA, Cat. #AV42783-50UG), rabbit polyclonal anti-CALHM1 (Alomone Labs, Israel, Cat. #ACC-101), rabbit polyclonal anti-eNOS (BD Transduction Laboratories™, USA, Cat. #610,298), mouse monoclonal anti-eNOS (BD Transduction Laboratories™, USA, Cat. #610,296), rabbit monoclonal anti-nNOS (Cell Signaling Technology, Cat. #4231), rabbit polyclonal anti-iNOS (Millipore, Germany, Cat. #ab5383), rabbit polyclonal anti-GFAP (Sigma Aldrich, USA, Cat. #G9269), mouse monoclonal anti-GFAP (Sigma Aldrich, USA, #G3893), rabbit polyclonal anti-β-actin (Sigma Aldrich, USA, Cat. #A3853) and rabbit polyclonal anti-SNO-Cys (Sigma Aldrich, USA, Cat. #N5411).

### Chemicals

All chemicals of analytical grade were obtained from Merck (Darmstadt, Germany). ODQ, MOPS, BSA, and glutamate were purchased from Sigma-Aldrich (St. Louis, MO, USA). PPADS and t-ACPD were obtained from Tocris Bioscience (Bristol, UK); SNAP and RuR from Calbiochem (La Jolla, CA, USA); ^10^Panx and ^37,43^Gap27 from Genscript (Israel). SNAP was dissolved in dimethyl sulfoxide (DMSO), and then, was diluted in buffer solution to reach the final working concentration. The vehicle of SNAP did not have effect per se (data not shown).

### Statistical analysis

Values are represented as mean ± standard error. Comparisons between groups were made using paired or unpaired Student’s t-test, one-way ANOVA plus Bonferroni post hoc test, or two-way ANOVA as appropriate. P < 0.05 was considered significant.

### Supplementary Information

**Additional file 1**. The Additional files includes ten Supplementary Figures in which complementary analyses of NOS isoforms expression by Western blot and the increase in ethidium uptake rate activated by glutamate are shown. In addition, the participation of ATP in the propagation of Ca^2+^ waves, CALHM1 cellular distribution in astrocyte cultures and intact brain, the effect of L-NA on glutamate- or t-ACPD-induced ATP release and the increase in [Ca^2+^]_i_ evoked by SNAP are depicted. These figures also illustrate the magnitude of glutamate-elicited Ca^2+^ signaling observed in the presence of PPADS, ^37,43^Gap27 or ^10^Panx and the immunofluorescence analysis depicting the astrocyte selection process in primary cultures of brain cortex. Furthermore, the uncropped images of the Western blots of CALHM1, Cx43 and Panx-1 performed in primary cultures of astrocytes treated with a control siRNA or with a siRNA designed to inhibit the expression of CALHM1 protein are presented. **Figure S1.** Expression of nitric oxide synthase (NOS) isoforms in primary cultures of brain cortex astrocytes. The presence of the different isoforms of NOS was evaluated by Western blot analysis in three independent astrocytes cultures. Note that the expression of the isoforms endothelial NOS (eNOS) and neuronal NOS (nNOS) was clearly observed, but, in contrast, consistent with the immunofluorescence analysis (Figure 3), the signal for the inducible NOS (iNOS) isoform was not detected by Western blot. **Figure S2.** Glutamate triggers the activation of Cx hemichannels and Panx-1 channels in astrocytes through a mechanism mediated by the endothelial isoform of nitric oxide synthase. The activation of Cx hemichannels and Panx-1 channels was evaluated through the analysis of the increase in ethidium uptake rate observed in primary cultures of astrocytes in response to 10 µM glutamate in control conditions and in the presence of the mimetic peptides ^37,43^Gap27 (100 μM) or ^10^Panx (100 μM) or the NOS inhibitor N^ω^-nitro-L-arginine (L-NA, 100 μM). The peptide ^37,43^Gap27 is a blocker of hemichannels formed by Cx37 or Cx43, ^10^Panx is an inhibitor of the channels formed by Panx-1 and L-NA is a general blocker of the enzyme nitric oxide synthase. In addition, the increment in ethidium uptake rate activated by glutamate in control conditions and after the treatment with 60 nM N^ω^-Propyl-L-Arginine (N^ω^-Propyl-L Arg) is also shown. N^ω^-Propyl-L-Arg is a selective inhibitor of the neuronal nitric oxide synthase isoform. The rate of ethidium uptake was assessed by calculating the slope of the increase in fluorescence intensity (expressed as arbitrary units, AU) along the time in basal conditions and during the stimulation with glutamate. Numbers inside the bars indicate the n value. Values are means ± SEM. *, P<0.05 vs the response to glutamate in Control by one-way ANOVA plus Bonferroni post hoc test. **Figure S3.** Astrocytic Ca^2+^ signaling activated by mechanical stimulation-mediated ATP release depends on the opening of hemichannel. To confirm the participation of hemichannels in ATP-initiated Ca^2+^ signaling in primary cultures of brain cortex astrocytes, a single cell was mechanically stimulated to trigger endogenous release of ATP. Recently, direct measurements of ATP in the vicinity of the stimulated cell demonstrated that single cell mechanical stimulation elicits ATP release through P2X7 channels in a Ca^2+^-independent manner (Xiong et al., J. Physiol., 596.10: 1931–1947, 2018, https://doi.org/https://doi.org/10.1113/JP275805). Single cell mechanical stimulation was applied with the tip of a polished micropipette (~ 5 µm), which was carefully moved down using a micromanipulator (Burleigh TS-5000-I50) to deliver a slight touch on the cell surface. As Ca^2+^ signaling can be transmitted directly via gap junctions from the stimulated cell to adjacent astrocytes, changes in [Ca^2+^]_i_ were analyzed in the stimulated cell (St Cell, black bar) and at two cells of distance (~ 100 µm) from the stimulation site (distant astrocytes, white bars) in control conditions (Control) and in the presence of 100 µM pyridoxalphosphate-6-azophenyl-2',4'-disulfonic acid (PPADS), 100 µM ^43^Gap26 or 50 µM 18βglycyrrhetinic acid (β-GA), as shown in the representative images (left). The peptide ^43^Gap26 was applied 5 min before the stimulation to only block hemichannels, without affecting the activity of gap junction channels. As expected, the increase in [Ca^2+^]_i_ observed in distant astrocytes was blocked by PPADS, corroborating the participation of ATP in the response. In addition, consistent with the involvement of Cx43 hemichannels in the increase of [Ca^2+^]_i_ induced by exogenous application of 100 nM ATP (Figure 5D), Ca^2+^ signals of distant astrocytes were also inhibited by the Cx inhibiting peptide ^43^Gap26 and the general blocker of Cx-formed channels, β-GA (Right). Note that changes in the Ca^2+^ signaling observed in the presence of PPADS, ^43^Gap 26 and β-GA are not significantly different. The treatment with PPADS, ^43^Gap26 or β-GA did not affect the increase in [Ca^2+^]_i_ triggered directly by mechanical stimulation. Arrows indicate the stimulated cell. Numbers inside the bars indicate the n value (total number of cells analyzed in three or more independent cell cultures). Values are means ± SEM. *, P < 0.05 vs Control by one-way ANOVA plus Bonferroni post hoc test. **Figure S4.** The release of ATP evoked by activation of mGluR in primary cultures of brain cortex astrocytes depends on NO production. ATP release was measured 3 min after the stimulation with glutamate or t-ACPD in control conditions and in the presence of 100 µM Ν^ω^nitro-L-arginine (L-NA), an inhibitor of NO production. The effect of the vehicle of glutamate or t-ACPD is also shown. Numbers inside the bars indicate the n value. Values are means ± SEM. *, P < 0.05 vs Vehicle by one-way ANOVA plus Bonferroni post hoc test. **Figure S5.** Expression of CALHM1 in primary cultures of astrocytes. The expression of CALHM1 (red) was detected by immunofluorescence analysis in primary cultures of astrocytes. The cell nuclei are highlighted by the staining with DAPI (blue). **Figure S6.** Detection of CALHM1 in astrocytes of intact brain. The expression of CALHM1 (red) in astrocytes was detected by co-immunofluorescence analysis with glial fibrillary acidic protein (GFAP, green), an astrocyte marker. The combination of the GFAP and CALHM1 signals is also shown (Merge) and the resulting yellow signal attests to the expression of CALHM1 in astrocytes. In addition, higher magnification of the boxed area depicted in the merged images of GFAP and CALHM1 is shown in the upper right corner. In these experiments, anaesthetized male Sprague-Dawley rats (230-250 g) were perfused through the left ventricle with a PBS solution kept at 37° C to wash out the blood by an incision in the right atrium, and, immediately after, with a Bouin solution for 10 min to fix the tissues. Thus, rats were decapitated, the brains were rapidly isolated and post-fixed for 24 h. Brains were dehydrated, embedded in paraffin, sectioned (10 µm), placed on charge-coated slides and deparaffinized using standard procedures. The sections were blocked with 0.5% BSA in PBS, incubated with a rabbit polyclonal primary antibody anti-CALHM1 (Alomone Labs, Israel) and a mouse monoclonal anti-GFAP (Sigma Aldrich, USA), and then, with an Alexa-568-labeled goat anti-rabbit secondary antibody and an Alexa-488-labeled goat anti-mouse secondary antibody (Invitrogen Molecular Probes, USA) using the Signal Enhancer HIKARI (Nacalai Tesque, INC, Japan) as indicated by the manufacturer. The fluorescent signal was examined using an Olympus BX41 WI microscope and a CCD camera (Jenoptik ProgRes C5). **Figure S7.** Uncropped images of Western blots shown in main Figure 6. Red box areas indicate the cropped regions used as representatives in Figure 6B (CALHM1), 6C (Cx43) and 6D (Panx-1). The first lane of each Western blot was loaded with a sample of primary cultures of astrocytes treated with a control siRNA (siControl) and the second lane with a sample of primary cultures of astrocytes treated with a siRNA designed to inhibit the expression of CALHM1 protein (siCALHM1). Membranes were first probed for CALHM1 (upper panel), Cx43 (middle panel) or Panx-1 (lower panel), and then, stripped to detect β-actin, as load control. **Figure S8.** NO signaling leads to the activation of an increase in [Ca^2+^]_i_ in primary cultures of brain cortex astrocytes. Maximal increment in [Ca^2+^]_i_ elicited by the stimulation with 3 µM SNAP, a NO donor, in control

## Data Availability

The datasets used and/or analysed during the current study are available from the corresponding author on reasonable request.

## Abbreviations

Cx43: Connexin-43
Panx1: Pannexin-1
CALHM1: Calcium homeostasis modulator 1
[Ca^2+^]_i_: Intracellular calcium concentration
mGluR: Metabotropic glutamate receptor
NO: Nitric oxide
ATP: Adenosine 5′-triphosphate
NOS: Nitric oxide synthase
eNOS: Endothelial nitric oxide synthase
nNOS: Neuronal nitric oxide synthase
iNOS: Inducible nitric oxide synthase
t-ACPD: Trans-1-amino-1,3-cyclopentanedicarboxylic acid
L-NA: N^ω^-nitro-L-arginine
RuR: Ruthenium red
PPADS: Pyridoxalphosphate-6-azophenyl-2′,4′-disulfonic acid
SNAP: S-nitroso-N-acetylpenicillamine
