# Supplementary material for: Control of astrocytic Ca2+ signaling by nitric oxide-dependent S-nitrosylation of Ca2+ homeostasis modulator 1 channels
Source: Biol Res. 2024 Apr 30;57:19. doi: 10.1186/s40659-024-00503-3 (PMC11059852; doi:10.1186/s40659-024-00503-3)
Supplement: Supplementary file 1 — Additional file 1. The Additional files includes ten Supplementary Figures in which complementary analyses of NOS isoforms expression by Western blot and the increase in ethidium uptake rate activated by glutamate are shown. In addition, the participation of ATP in the propagation of Ca2+ waves, CALHM1 cellular distribution in astrocyte cultures and intact brain, the effect of L-NA on glutamate- or t-ACPD-induced ATP release and the increase in [Ca2+]i evoked by SNAP are depicted. These figures also illustrate the magnitude of glutamate-elicited Ca2+ signaling observed in the presence of PPADS, 37,43Gap27 or 10Panx and the immunofluorescence analysis depicting the astrocyte selection process in primary cultures of brain cortex. Furthermore, the uncropped images of the Western blots of CALHM1, Cx43 and Panx-1 performed in primary cultures of astrocytes treated with a control siRNA or with a siRNA designed to inhibit the expression of CALHM1 protein are presented. Figure S1. Expression of nitric oxide synthase (NOS) isoforms in primary cultures of brain cortex astrocytes. The presence of the different isoforms of NOS was evaluated by Western blot analysis in three independent astrocytes cultures. Note that the expression of the isoforms endothelial NOS (eNOS) and neuronal NOS (nNOS) was clearly observed, but, in contrast, consistent with the immunofluorescence analysis (Figure 3), the signal for the inducible NOS (iNOS) isoform was not detected by Western blot. Figure S2. Glutamate triggers the activation of Cx hemichannels and Panx-1 channels in astrocytes through a mechanism mediated by the endothelial isoform of nitric oxide synthase. The activation of Cx hemichannels and Panx-1 channels was evaluated through the analysis of the increase in ethidium uptake rate observed in primary cultures of astrocytes in response to 10 µM glutamate in control conditions and in the presence of the mimetic peptides 37,43Gap27 (100 μM) or 10Panx (100 μM) or the NOS inhibitor Nω-nitr [file 40659_2024_503_MOESM1_ESM.pdf]

Additional file 1

Control of astrocytic Ca^{2+} signaling by nitric oxide-dependent S-nitrosylation of Ca^{2+} homeostasis modulator 1 channels

Mariela Puebla[1]; Manuel Muñoz[1,2]; Mauricio A Lillo[3]; Jorge Contreras[2,3]; Xavier F. Figueroa[1*]

[1]Departamento de Fisiología, Facultad de Ciencias Biológicas, Pontificia Universidad Católica de Chile, Santiago 8330025, Chile.

[2]Department of Physiology and Membrane Biology, University of California Davis, Davis, CA, United States.

[3]Department of Pharmacology, Physiology and Neuroscience, New Jersey Medical School, Rutgers University, Newark, NJ, United States.

Running title: NO/CALHM1 signaling in astrocyte Ca^{2+}

***Author for correspondence:**

Dr. Xavier F. Figueroa

Departamento de Fisiología

Facultad de Ciencias Biológicas

Pontificia Universidad Católica de Chile

Santiago, Chile

Phone: 562-2686-2356

E-mail: xfigueroa@bio.puc.cl

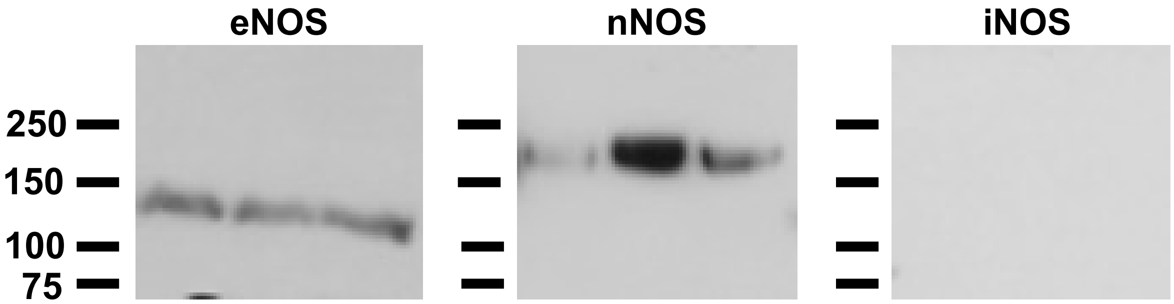

Figure S1. Expression of nitric oxide synthase (NOS) isoforms in primary cultures of brain cortex astrocytes. The presence of the different isoforms of NOS was evaluated by Western blot analysis in three independent astrocytes cultures. Note that the expression of the isoforms endothelial NOS (eNOS) and neuronal NOS (nNOS) was clearly observed, but, in contrast, consistent with the immunofluorescence analysis (Fig. 3), the signal for the inducible NOS (iNOS) isoform was not detected by Western blot.

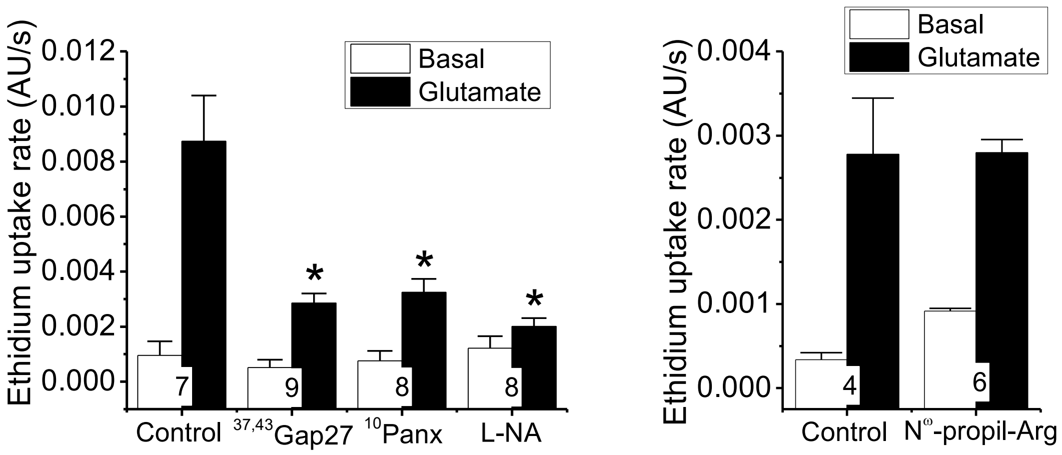

Figure S2. Glutamate triggers the activation of Cx hemichannels and Panx-1 channels in astrocytes through a mechanism mediated by the endothelial isoform of nitric oxide synthase. The activation of Cx hemichannels and Panx-1 channels was evaluated through the analysis of the increase in ethidium uptake rate observed in primary cultures of astrocytes in response to 10 μM glutamate in control conditions and in the presence of the mimetic peptides ^{37,43}Gap27 (100 μM) or ^{10}Panx (100 μM) or the NOS inhibitor N$^{\omega}$-nitro-L-arginine (L-NA, 100 μM). The peptide ^{37,43}Gap27 is a blocker of hemichannels formed by Cx37 or Cx43, ^{10}Panx is an inhibitor of the channels formed by Panx-1 and L-NA is a general blocker of the enzyme nitric oxide synthase. In addition, the increment in ethidium uptake rate activated by glutamate in control conditions and after the treatment with 60 nM N$^{\omega}$-Propyl-L-Arginine (N$^{\omega}$-Propyl-L-Arg) is also shown. N$^{\omega}$-Propyl-L-Arg is a selective inhibitor of the neuronal nitric oxide synthase isoform. The rate of ethidium uptake was assessed by calculating the slope of the increase in fluorescence intensity (expressed as arbitrary units, AU) along the time in basal conditions and during the stimulation with glutamate. Numbers inside the bars indicate the n value. Values are means ± SEM. *, $P<0.05$ vs the response to glutamate in Control by one-way ANOVA plus Bonferroni post hoc test.

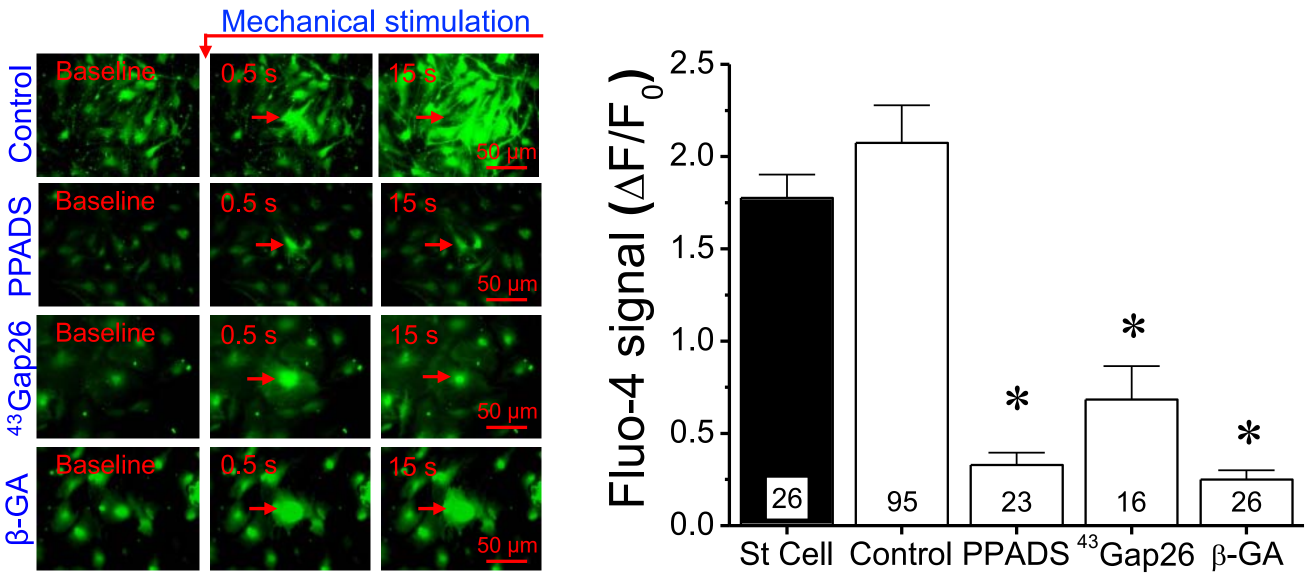

Figure S3. Astrocytic Ca^{2+} signaling activated by mechanical stimulation-mediated ATP release depends on the opening of hemichannel. To confirm the participation of hemichannels in ATP-initiated Ca^{2+} signaling in primary cultures of brain cortex astrocytes, a single cell was mechanically stimulated to trigger endogenous release of ATP. Recently, direct measurements of ATP in the vicinity of the stimulated cell demonstrated that single cell mechanical stimulation elicits ATP release through P2X7 channels in a Ca^{2+}-independent manner (Xiong et al., J. Physiol., 596.10: 1931–1947, 2018, DOI: 10.1113/JP275805). Single cell mechanical stimulation was applied with the tip of a polished micropipette (~5 µm), which was carefully moved down using a micromanipulator (Burleigh TS-5000-I50) to deliver a slight touch on the cell surface. As Ca^{2+} signaling can be transmitted directly via gap junctions from the stimulated cell to adjacent astrocytes, changes in $[Ca^{2+}]_i$ were analyzed in the stimulated cell (St Cell, black bar) and at two cells of distance (~100 µm) from the stimulation site (distant astrocytes, white bars) in control conditions (Control) and in the presence of 100 µM pyridoxalphosphate-6-azophenyl-2',4'-disulfonic acid (PPADS), 100 µM ^{43}Gap26 or 50 µM 18β-glycyrrhetinic acid (β-GA), as shown in the representative images (left). The peptide ^{43}Gap26 was applied 5 min before the stimulation to only block hemichannels, without affecting the activity of gap junction channels. As expected, the increase in $[Ca^{2+}]_i$ observed in distant astrocytes was blocked by PPADS, corroborating the participation of ATP in the response. In addition, consistent with the involvement of Cx43 hemichannels in the increase of $[Ca^{2+}]_i$ induced by exogenous application of 100 nM ATP (Fig. 5D), Ca^{2+} signals of distant astrocytes were also inhibited by the Cx inhibiting peptide ^{43}Gap26 and the general blocker of Cx-formed channels, β-GA (Right). Note that changes in the Ca^{2+} signaling observed in the presence of PPADS, ^{43}Gap 26 and β-GA are not significantly different. The treatment with PPADS, ^{43}Gap26 or β-GA did not affect the increase in $[Ca^{2+}]_i$ triggered directly by mechanical stimulation. Arrows indicate the stimulated cell. Numbers inside the bars indicate the n value (total number of cells analyzed in three or more independent cell cultures). Values are means ± SEM. *, $P<0.05$ vs Control by one-way ANOVA plus Bonferroni post hoc test.

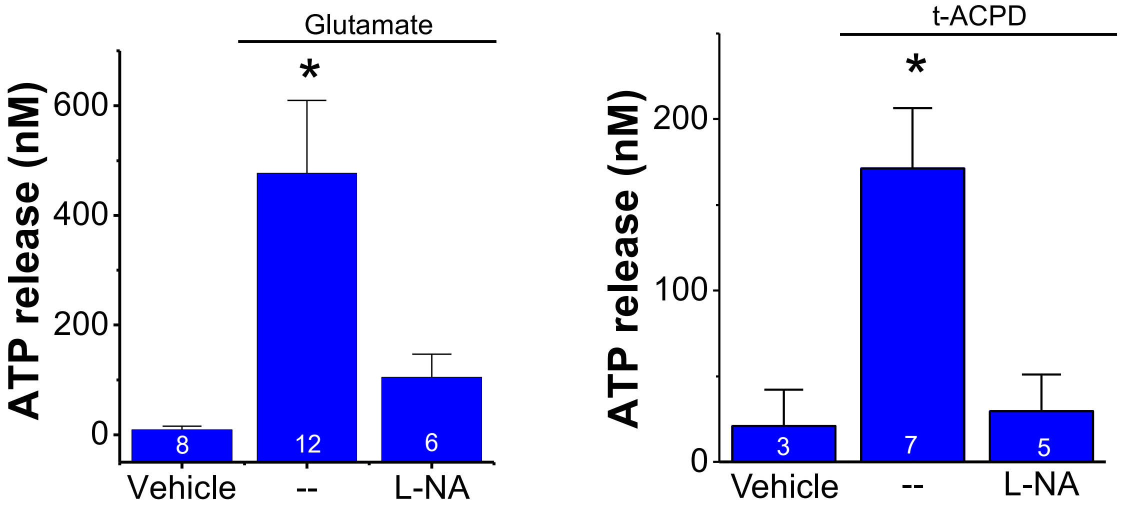

Figure S4. The release of ATP evoked by activation of mGluR in primary cultures of brain cortex astrocytes depends on NO production. ATP release was measured 3 min after the stimulation with glutamate or t-ACPD in control conditions and in the presence of 100 μM N^{ω}-nitro-L-arginine (L-NA), an inhibitor of NO production. The effect of the vehicle of glutamate or t-ACPD is also shown. Numbers inside the bars indicate the n value. Values are means ± SEM. *, $P<0.05$ vs Vehicle by one-way ANOVA plus Bonferroni post hoc test.

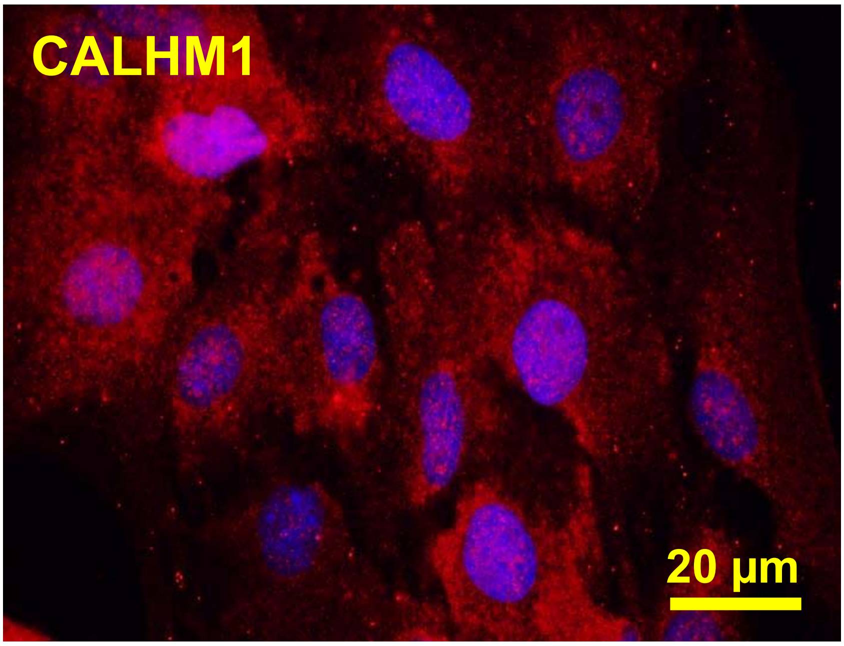

Figure S5. Expression of CALHM1 in primary cultures of astrocytes. The expression of CALHM1 (red) was detected by immunofluorescence analysis in primary cultures of astrocytes. The cell nuclei are highlighted by the staining with DAPI (blue).

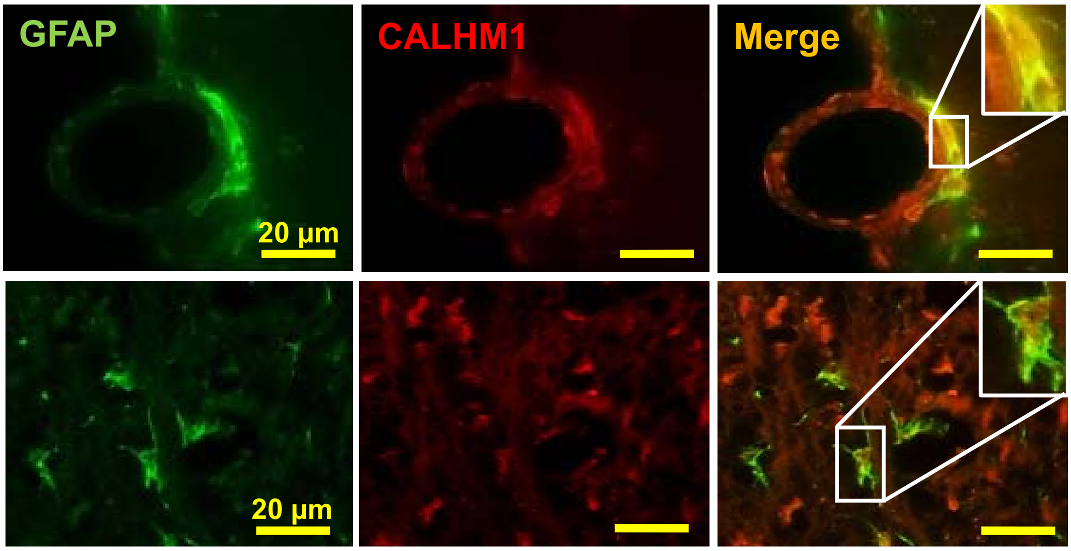

Figure S6. Detection of CALHM1 in astrocytes of intact brain. The expression of CALHM1 (red) in astrocytes was detected by co-immunofluorescence analysis with glial fibrillary acidic protein (GFAP, green), an astrocyte marker. The combination of the GFAP and CALHM1 signals is also shown (Merge) and the resulting yellow signal attests to the expression of CALHM1 in astrocytes. In addition, higher magnification of the boxed area depicted in the merged images of GFAP and CALHM1 is shown in the upper right corner. In these experiments, anaesthetized male Sprague-Dawley rats (230-250 g) were perfused through the left ventricle with a PBS solution kept at 37° C to wash out the blood by an incision in the right atrium, and, immediately after, with a Bouin solution for 10 min to fix the tissues. Thus, rats were decapitated, the brains were rapidly isolated and post-fixed for 24 h. Brains were dehydrated, embedded in paraffin, sectioned (10 µm), placed on charge-coated slides and deparaffinized using standard procedures. The sections were blocked with 0.5% BSA in PBS, incubated with a rabbit polyclonal primary antibody anti-CALHM1 (Alomone Labs, Israel) and a mouse monoclonal anti-GFAP (Sigma Aldrich, USA) , and then, with an Alexa-568-labeled goat anti-rabbit secondary antibody and an Alexa-488-labeled goat anti-mouse secondary antibody (Invitrogen Molecular Probes, USA) using the Signal Enhancer HIKARI (Nacalai Tesque, INC, Japan) as indicated by the manufacturer. The fluorescent signal was examined using an Olympus BX41 WI microscope and a CCD camera (Jenoptik ProgRes C5).

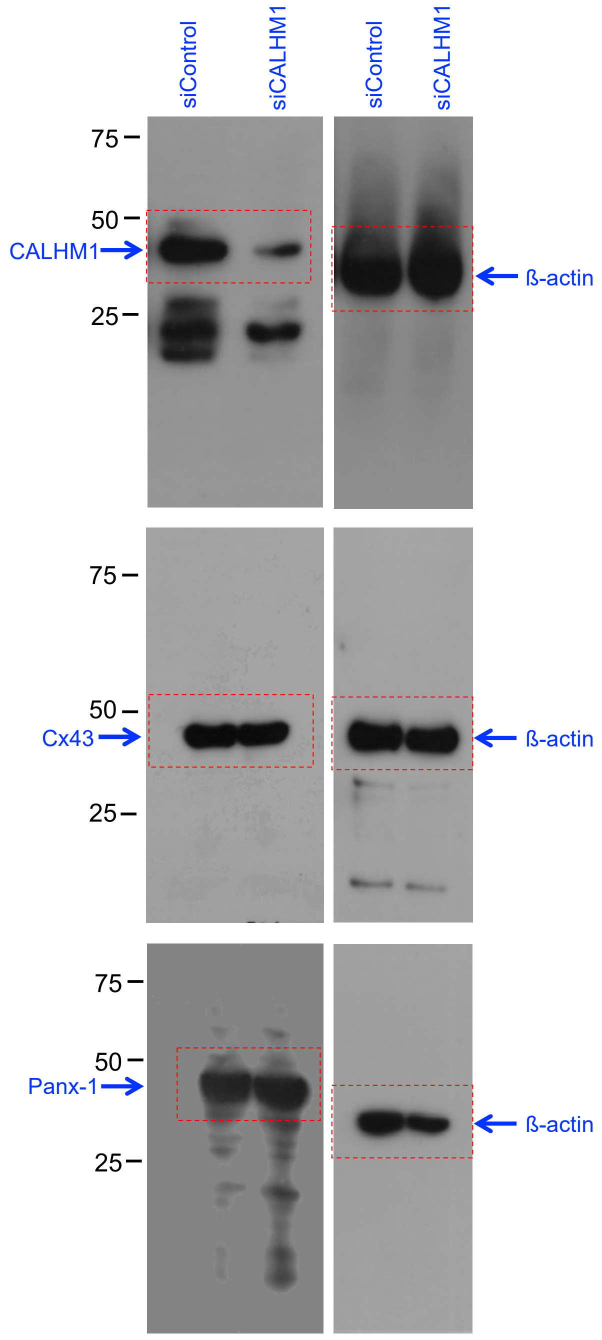

Figure S7. Uncropped images of Western blots shown in main Figure 6. Red box areas indicate the cropped regions used as representatives in Figure 6B (CALHM1), 6C (Cx43) and 6D (Panx-1). The first lane of each Western blot was loaded with a sample of primary cultures of astrocytes treated with a control siRNA (siControl) and the second lane with a sample of primary cultures of astrocytes treated with a siRNA designed to inhibit the expression of CALHM1 protein (siCALHM1). Membranes were first probed for CALHM1 (upper panel), Cx43 (middle panel) or Panx-1 (lower panel), and then, stripped to detect β-actin, as load control.

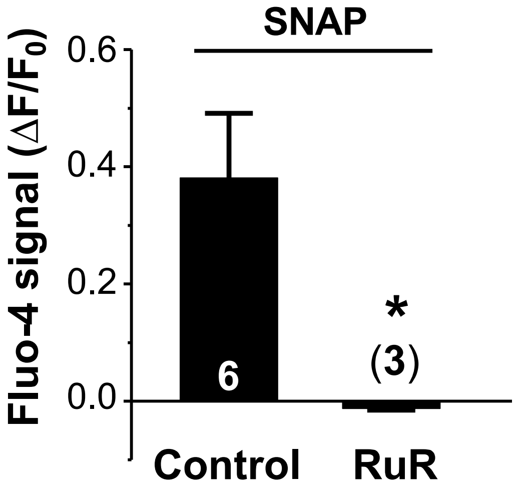

Figure S8. NO signaling leads to the activation of an increase in $[Ca^{2+}]_i$ in primary cultures of brain cortex astrocytes. Maximal increment in $[Ca^{2+}]_i$ elicited by the stimulation with 3 μM SNAP, a NO donor, in control conditions and in the presence of 20 μM ruthenium red (RuR) is shown. Note that blockade of CALHM1 channels with RuR abolished the Ca^{2+} response triggered by the activation of a NO-initiated signaling pathway. Numbers inside the bars or in parentheses indicate the n value. Values are means ± SEM. *, $P<0.05$ vs Control by unpaired Student's t-test.

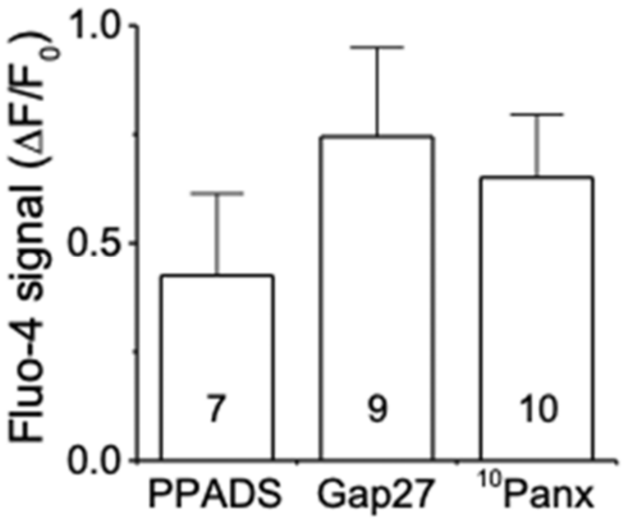

Figure S9. ATP signaling-independent increase in $[Ca^{2+}]_i$ induced by glutamate in primary cultures of brain cortex astrocytes. Maximal increment in $[Ca^{2+}]_i$ elicited by 10 μM glutamate in the presence of 100 μM PPADS, 100 μM ^{37,43}Gap 27 (Gap27) or 100 μM ^{10}Panx. Note that the changes in the Ca^{2+} signaling observed in all three conditions are not significantly different. Numbers inside the bars indicate the n value. Values are means ± SEM.

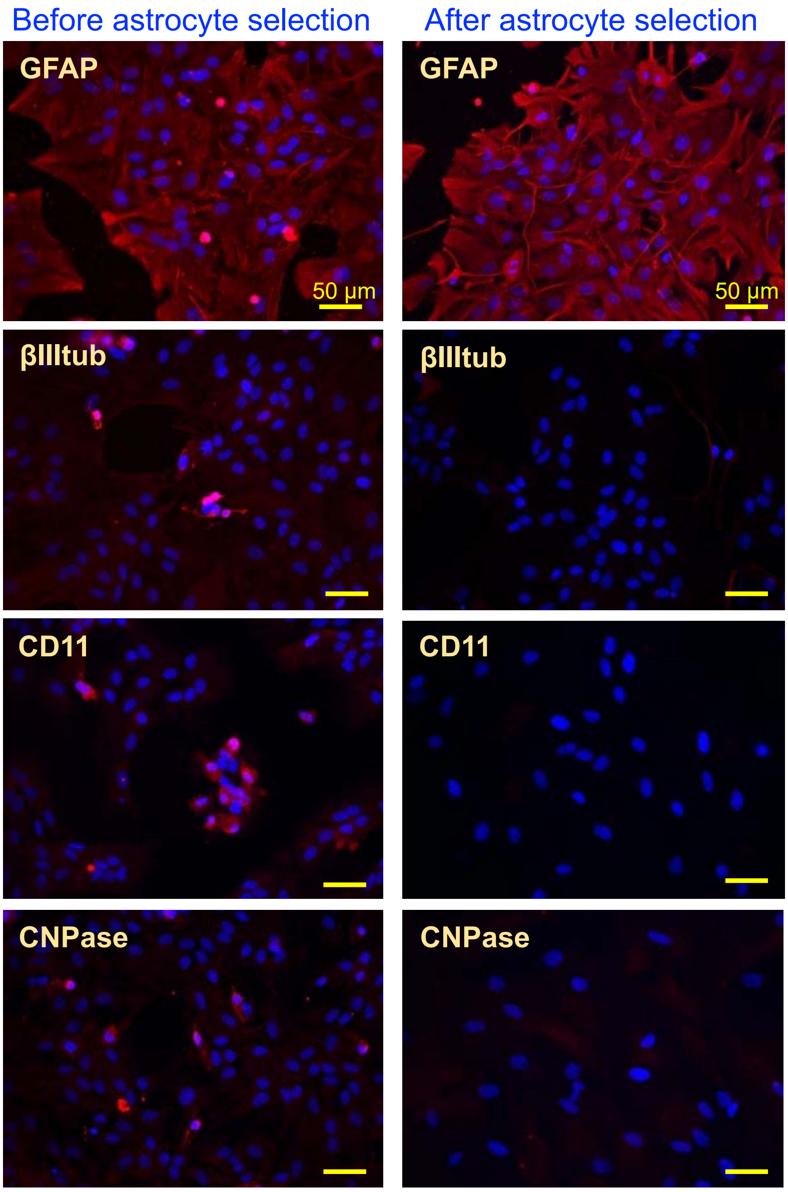

Figure S10. Immunofluorescence analysis depicting the astrocyte selection process in primary cultures of brain cortex. Primary cultures of astrocytes were prepared from brain cortex of 1-to-2-day-old neonatal rats, free of meninges. Cells were grown for ~1 week to reach a confluence of ~80%, and thus, the culture of mixed cortical cells was shaken (200 rpm) for 20 h at 37°C to remove neurons, microglia and oligodendrocytes, but keeping the remaining adherent astrocytes. The enrichement of astrocyte was confirmed through immunofluorescence staining by detecting the presence of GFAP, an astrocyte marker; ßIII tubulin (ßIIItub), a neuron marker; CD11, a microglia marker and CNPase, an oligodendrocyte marker, before and after the astrocyte selection process. The cell nuclei are highlighted by the staining with DAPI (blue). An astrocyte purity of 96±1.1% was determined by the analysis of three independent cultures.
